# G-protein coupled receptor, signal and signal-transduction related transcript mapping in nigral volume and pigmented neurons reveals a potential deficit of nigral feedback signals associated with Parkinson’s disease

**DOI:** 10.1371/journal.pone.0352503

**Published:** 2026-07-24

**Authors:** John M. Haynes

**Affiliations:** Drug Discovery Biology, Faculty of Pharmacy and Pharmaceutical Sciences, Monash University, Parkville, Victoria, Australia; University of North Texas Health Science Center, UNITED STATES OF AMERICA

## Abstract

Microarray and Next Generation Sequencing studies offer insight into gene regulation in Parkinson’s disease (PD). However, analysing vast numbers of genes can make the interpretation of data difficult when considering the platforms and techniques used across different studies. In this study, transcript expression, restricted to genes related to G-protein coupled receptors, activating agonists, agonist precursors, synthesis enzymes and transduction processes, as well as stress-related chaperones and solute carriers, was assessed across nine microarray platforms and two RNAseq studies of the substantia nigra (nigral volume). Changes in gene expression associated with PD were assessed by differential expression analysis while RNAseq studies were also used to calculate transcript per million values for each of the genes within the nigral volume. This analysis showed extensive changes in nigral volume signalling for several robustly expressed signal-related transcripts including tyrosine hydroxylase (*TH*), WNT signalling components (*SFRP1*, *RSPO2* and *DKK3*), Kallikrein Related Peptidase 6 (*KLK6*), neurexins (*NRXN1*, *NRXN3*), prostaglandin synthases (*PTGES2*, *PTGES3*) and fractalkine (*CX3CL1*). The nigral volume signalling ligand data was then cross-referenced to the most abundant G-protein coupled receptor and signal transduction transcripts in the pigmented neurons using two RNAseq and two microarray studies. There were 32 significant changes in G-protein coupled receptor and associated signalling genes in pigmented neurons from PD tissue. Of these, 30 genes were upregulated. Analysis of the transcription factors likely regulating the 30 upregulated genes indicates a stimulation of cell stress pathways, particularly the JNK-MAP kinase pathway. Following this pathway back to changes in nigral signalling transcripts indicates that deficits in at least two autocrine/paracrine signalling systems; dopamine, via Gα_o_-coupled dopamine D_2_ receptors and Dickkopf-3 (DKK3) appear likely to contribute to pigmented neuron stress in PD.

## Introduction

Parkinson’s disease (PD) affects 1–2% of individuals over 50 years of age [1]. These individuals suffer a variety of well-documented motor and non-motor symptoms. The motor symptoms include hypokinesia, muscle rigidity and shaking tremor [2], while the non-motor symptoms include depression, apathy, anxiety, hallucinations, sleep disorders, urinary urgency, nocturia, sexual dysfunction, dysphagia, fecal incontinence and paresthesia [3]. The classically described motor symptoms of Parkinson’s disease are accepted to result from the loss of tyrosine hydroxylase (TH) positive dopaminergic (A9) neurons of the substantia nigra pars compacta. This results in a deficit of striatal dopamine and impaired motor function. The basis of this understanding of Parkinson’s disease owes a great deal to early reports of organophosphate and illicit drug manufacture/ poisoning in the 1970s and early 1980s [4–6]. From this time the clinical symptoms of Parkinson’s disease were intertwined with the survival of the relatively small population of nigral A9 dopaminergic neurons.

Next-generation sequencing (NGS) and microarray studies have been instrumental in characterizing the transcriptomic changes evident in the substantia nigra of individuals with PD. These works include both studies of gene expression in whole tissues [7–13], as well as studies utilizing captured pigmented (neuromelanin-positive) neurons [11,14,15]. While microarray studies employ differential expression analysis (DEA) to show changes in gene expression as a ratio of change (PD/control), NGS offers a little more flexibility as it enables the calculation of DEA as well as quantitation of transcript; expressed for example, as transcript per million (TPM) values. The ability to quantitate transcript adds value to DEA analysis as it indicates the importance a cell is likely to place on having an abundance of a particular transcript. In this study, transcript DEA and TPM values were determined within the substantia nigra (pars compacta), pars compacta pigmented neurons and the striatum (both caudate nucleus and putamen), which serves as both a target for dopaminergic neurons and a source of feedback signalling [16]. Of course, having an abundance of a transcript does not necessarily mean an abundance of a protein, but until protein levels are absolutely determined, transcript levels seem a reasonably suitable surrogate.

The purpose of this study is to map changes in G-protein coupled receptor ligand or ligand-related transcripts, both locally within the substantia nigra and from likely striatal inputs. These data are then matched to equivalent G-protein coupled receptor and related signal transduction pathway transcript expression in pigmented neurons to establish how PD-related changes in nigral signalling might impact neuron signal transduction. This work now identifies major PD-related changes in local nigral volume signalling including expected major reductions in transcripts for dopa decarboxylase (*DDC*) and tyrosine hydroxylase (*TH*) but also changes in the WNT signalling modulators; Dickkopf-3 (*DKK3*), roof plate specific spondin-2 (*RSPO2*) as well as multiple changes in peptide, lipid, glycoprotein (fractalkine) and prostaglandin signalling systems. The two most highly expressed receptor transcripts in the pigmented neurons are for the Gα_S_ coupled prostacyclin receptor and the predominantly Gα_i/o_ coupled dopamine D_2_ receptor. This appears consistent with the pigmented neurons showing an abundance of Gα_S_ and Gα_o1_ transcripts. While Gα_S_ signalling appears to remain relatively intact in PD, Gα_o1_-related and WNT ligand modulated signal transduction appears, at least at the level of transcript, impaired. The implications of potentially reduced WNT and Gα_o_ signalling on dopaminergic neuron function and survival are discussed within the context of cellular signalling pathways.

## Materials and methods

This study utilized microarray and RNA seq PD databases including whole substantia nigra, putamen and caudate nucleus tissue as well as studies using isolated pigmented neurons: GSE8397, GSE7621, GSE7307, GSE43490, GSE42966, GSE20333, GSE20292, GSE20164, GSE20163, GSE136666, GSE114517, GSE182622, GSE24378, GSE20141, GSE114918 and GSE205450 (**S1 Table**). These databases were assessed for changes in genes identified as (druggable) targets by the International Union of Pharmacologists/ British Journal of Pharmacology Guide to pharmacology (https://www.guidetopharmacology.org/ # GtoPdb Version: 2022.4). This study also used an ad hoc list of genes associated with soluble mediator-related signalling, solute carriers and stress-related proteins. Thus, for example, the signalling data set includes dopa decarboxylase and tyrosine hydroxylase (related to catecholaminergic signalling), WNTs, chemokines, angiotensin converting enzyme and prostaglandin synthases. **S2 Table** shows the full list of genes of interest (GOIs) used for isolated neurons, whole nigra, caudate nucleus and putamen studies.

### Differential gene expression analysis (DEA)

For microarray studies SOFT family formatted data (counts) and RNAseq study counts were downloaded (from https://www.ncbi.nlm.nih.gov/geo/query/acc.cgi). For differential expression analysis, data counts were standardized to the expression of a series of housekeeper genes (*GAPDH, ACTB, STAT1, HPRT1, GUSB, TBP, B2M, ACTN1, PPIA, PGK1, RPLP0, TFRC*), where available. Sometimes raw count PD vs control housekeeper genes were significantly different (Student’s t-test, not shown). These differences did not, however, form a pattern across the data sets. So, for example GSE7621 showed a significant PD versus control difference for *STAT1* while GSE8397 (medial) showed a significant difference for *PPIA* while GSE20164 showed no differences between any housekeeper genes.

After standardization to their respective housekeeper gene sets, PD and control data were ratioed. Where a study contained eight PD tissue samples and six control samples, then only six randomly assigned PD: control ratio sets were used; the remaining two PD samples ignored. This approach was adopted, instead of simply taking the mean of all PD samples over the mean of all control samples for each study, to assign appropriate value to studies with different “n” values. While the pairs of control and PD data were assigned at random, in circumstances where data sets recorded “0” values, the “0” values were ignored and ratio was created from the next available number. So, for example if PD and control values read: 0, 5, 0, 3 (PD counts) and 2, 0, 1, 0 (control counts) this translated to PD: control ratios of 5:2 and 3:1. Prior to hypothesis testing, univariate technical and extreme biological anomalies within the paired values were identified and removed utilizing the robust regression followed by outlier identification (ROUT) method (Q = 1%, GraphPad Software 10.1.2, Boston, Massachusetts USA, www.graphpad.com). Cleaned data were then expressed as log_2_ and subject to one-sided t-tests (tested against 0). Resulting P-values were stratified into discrete sample size (n-value) clusters to control for variation in statistical power. Only dataset GOI values with an “n” value greater than 45% of the total number of samples were assessed (n ≥ 37 for nigral tissue, n ≥ 14 for pigmented neurons and n ≥ 16 for putamen and caudate nucleus). The n value filtering step was undertaken to ensure that only genes with common platform probes or clear expression were included in the P value analysis. It should be noted that robust GOI expression is a factor dependent upon sample quality and quantity, cell expression, the tissue used (i.e., the region boundaries set by the investigators), the limitations of RNAseq coverage and depth and/or the microarray probe sets used. Once through filtering, the n-value stratified P values were analysed using the two-stage linear step-up procedure of Benjamini, Krieger and Yekutieli (GraphPad Software 10.1.2, Boston, Massachusetts USA, www.graphpad.com) to identify “discoveries”, with Q set at 3% (whole tissue studies) or 30% (pigmented neurons). The requirement for an increase in pigmented neuron Q value is consistent with the increased variability of the pigmented neuron data. **S1 Fig** shows a plot of TPM (RNAseq) vs DEA standard deviation (SD, combined RNAseq and microarray studies). There are two interesting observations from this data: (i) As TPM increases a one-phase decay curve fits the pigmented neuron SD data better than a linear regression (F-test, P < 0.0001), linear regression is the appropriate fit for the nigral volume data. (ii) An F-test shows significant differences in sample variances (P < 0.0001, F-test). Since data processing is identical, the increased variability of the pigmented neuron samples seems likely due to the limited starting material used for the individual studies. Thus, while the tissue studies assessed in this manuscript utilize hundreds of micrograms of RNA per platform, the pigmented neuron studies sample up to 200 pigmented neurons. As a guide, the total RNA used per platform is likely in the range of 5.4ng (based on an RNA per neuron (cortex) value of 27pg/neuron [17]). In the context of microarray and RNAseq studies, such small samples are very likely to lead to higher rates of amplification bias, distortion and increased background noise: signal [18–21]. Thus, the increased FDR Q threshold for pigmented neuron samples is an appropriate measure in the face of increased pigmented neuron sample variability.

Generally, the statistical approach used in this study is designed to filter out background noise, with the combination of ratiometric normalisation, random serialization, outlier removal and n-value tiered false discovery management likely to favour conservative and uniform changes in expression. This approach is likely to mean that the interpretation of data may be influenced by small changes in subsets of housekeepers masking small changes in GOI expression. Also, the random ratio pairing introduces noise into the analysis that may be mitigated somewhat by the ROUT filtering which may, inadvertently, exclude true PD-related changes against a background of inclusion with less severe disease phenotypes. Including multiple FDR analyses, stratified by “n” values might inflate the rate of false-positive biological discoveries in smaller n-value sub-analyses. Essentially this analysis will systematically filter out low-abundance or highly variable signalling pathways. Thus, the biological inferences are likely to be dependable, but limited to uniform, middle-to-high-abundance transcript changes. Lastly, this study will not identify GOIs where transcript levels increase to, or decrease from detectable levels since no DEA ratio can be formed.

### Transcript per million (TPM) analysis

The pigmented neuron RNAseq (GSE182622 and GSE114918) and the two substantia nigra RNAseq studies (GSE136666 and GSE114517) were used to estimate average transcript per million (TPM) values to define the relative abundance of transcripts within the pigmented neurons and substantia nigral volume. For this analysis, counts were normalized for gene length to get Reads Per Kilobase (RPK) then scaled by dividing RPKs by the sum of RPKs per million. Control data was subsequently logged_2_ and averaged. Where the DEA analysis indicated a significant change in expression associated with PD, the average control sample TPM expression is shown in the figures along with the DEA ratio adjusted control TPM. This approach was chosen to align with the unification of the combined microarray and RNAseq studies. An additional RNAseq study [22] that used caudate nucleus and putamen samples from control and individuals with PD was used for comparative purposes (GSE205450).

### ChEA3 analysis

ChEA3 is a transcription factor analysis tool that identifies transcription factor overlap within given lists of differentially expressed genes [23]. Both positively and negatively regulated, differentially expressed pigmented neuron GOIs were imported into ChEA3 for analysis to generate lists of transcription factors associated with GOI transcript expression.

## Results

### Pigmented neuron GPCR TPM & DEA analysis

Within the dopaminergic (pigmented) neuron population of the substantia nigra, distinct dorsal and ventral subsets exist. Among these, the ventral aldehyde dehydrogenase 1A1-positive (*ALDH1A1*) and calbindin 1-negative (*CALB1*) population exhibits the highest vulnerability in Parkinson’s disease (PD) (see [24]). The pigmented neurons collected for these studies show high expression of *ALDH1A1* transcript (807 TPM), with modest expression of calbindin transcript (*CALB1*) in control samples (3.6 TPM). That DEA analysis does not indicate change in PD samples establishes a consistent collection of pigmented neurons likely from the ventral substantia nigra pars compacta.

The expression of GPCR transcripts was assessed in pigmented neurons using a curated list of 402 receptor genes (https://www.guidetopharmacology.org/GRAC). Of these, 374 showed low expression (TPM < 5) or were undetectable, see **S3 Table**. Just looking at the top 10 GPCRs by TPM: the dopamine D_2_ receptor (*DRD2*) is the most abundant GPCR transcript (86 TPM), followed by the prostacyclin receptor (*PTGIR*, 46 TPM), neurotensin receptor (*NTSR1*, 39 TPM), GABA_B2_ receptor (*GABBR2*, 35 TPM), GABA_B1_ receptor (*GABBR1*, 25 TPM), the adhesion receptor, *ADGRL1* (23 TPM), the orphan *GPR162* (22 TPM), somatostatin receptor (*SSTR1*, 18 TPM), the orphan *GPR26* (15 TPM) and the chemokine scavenger, *ACKR1* (15 TPM). The top 10 GPCR transcripts represent 43% of the total GPCR transcripts within the pigmented neurons. Of these, only *SSTR1* showed a significant change (reduction) in expression (P = 0.00052), see **S2 Table**.

GPCRs can activate multiple signal transduction pathways commonly through Gα or Gβγ subunits or following β-arrestin-stimulated internalization. Generally, signal transduction pathways are defined through the families of G-protein alpha subunits activated, i.e. Gα_i/o_, Gα_s_, Gα_q_ and Gα_12/13_. To reduce some of the complexity associated with GPCR signal transduction the IUPHAR/BPS guide to pharmacology [25] was used to identify the primary G-protein transduction coupling mechanism. This approach enabled a grouping of pigmented neuron receptors by primary G-protein α subunit transduction mechanism for the 20 most highly expressed GPCRs (**S3 Table**). It is apparent that many of these highly expressed receptors have primary signal transduction mechanisms that negatively regulate the activity of adenylate cyclase (through the Gα_i/o_ family). The subsequent activation pathways are adapted from the review by Lappano and Maggiolini [26], although other sources are used (as indicated). To reduce repetition no attempt has been made to distribute receptors according to neuroanatomical location. Thus, somatodendritic and nerve terminal receptors are shown together with reference to only primary signal transduction processes. Similarly, different transcript processing variants, for example DRD2 short and long forms, are included together. Note too that ADGRL1 and AGTR1 appear highly promiscuous with respect to preferred or primary G-protein coupling (see **S3 Table**).

While GPCRs with primarily Gα_i/o_ family member coupling predominate within the pigmented neurons, Gα_s_ (*GNAS*) is by far the most abundant G-protein subunit transcript, followed by Gα_o1_ (*GNAO1*), Gα_q_ (*GNAQ*), Gα_i2_ (*GNAI2*), and Gα_11_ (*GNA11*) (**S2 Table**). Beyond the Gα subunits, *GNB1* and *GNB2* are the most abundant G-protein β subunit transcripts while *GNG3* is by far the predominant G-protein γ subunit transcript (**S2 Table**). Thus, it seems likely that most of the G-protein βγ subunits present in pigmented neurons are likely combinations of β_1_γ_3_ or β_2_γ_3_. Although it is unclear why there are far fewer Gβ subunit transcripts than Gα or Gγ.

### Gα_s_ coupled GPCR signalling

**Fig 1:**
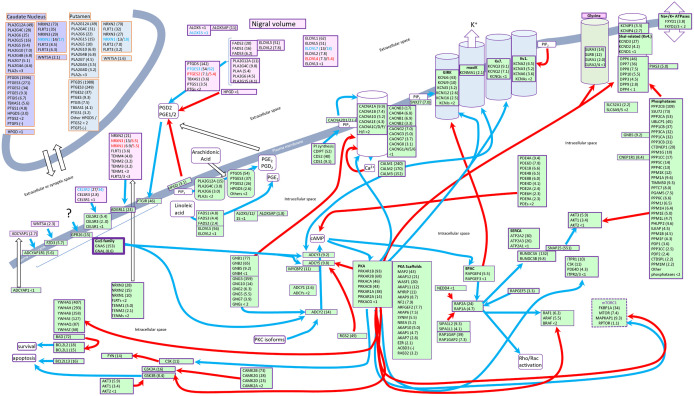
Speculative Gα_s_ family signalling pathways in pigmented neurons. The image includes gene symbols for transcripts for ligands presumed present in the nigral volume and/or synaptic spaces (pink boxes), putamen or caudate nucleus (mauve and blue boxes, respectively) and the pigmented neurons (green boxes). The numbers in the brackets represent the transcript per million (TPM) from control tissue/cells. If the differential expression analysis (DEA) of the data set shows a significant change transcript associated with Parkinson’s disease, then the gene name is coloured red for a decrease and blue for an increase. The significant change in expression (control TPM adjusted by DEA value) is represented by the number after the forward slash (/). The white boxes (for example, the one containing cAMP) generally indicate a process or substance produced, introduced into or activated within the intra- or extracellular space. The red arrows indicate an inhibitory action while the blue arrows indicate a stimulatory action (sometimes blue and red arrows connect the same pathways to indicate context dependent signalling). The white arrows indicate a potential for substance movement across membranes. When boxes are connected it indicates a likely binding partner (PKA and PKA scaffolds, for example). Some transcripts, for example some of the phosphatases, are weakly expressed and therefore listed as “Other phosphatases <2”, indicating that remaining family member transcripts are expressed at less than 2 TPM (**S2 Table** shows all transcript values). This is a stylized map of pathway activity it is not intended to define subcellular or extracellular locations of transcript derived proteins. See text for details of major pathway interactions and definitions.

Within the pigmented neurons, Gα_s_ is by far the most abundantly expressed G-protein α-subunit (**S2 Table**). Within the top 20 most highly expressed GPCR transcripts there are three GPCRs that are indicated to have primary Gα_s_ coupling: *PTGIR*, *ADGRL1* and *GPR26.* There are also two GPCRs that appear to have possible Gα_s_ coupling, frizzled-3 (*FZD3*) and the Cadherin EGF LAG Seven-Pass G-Type Receptor 2 (*CELSR2*) [27]. CELSR2 has also been suggested to drive WNT-β-catenin signalling [28] although this effect may, however, be mediated via increased protein kinase A activation [29]. Given the high levels of pigmented neuron Gα_s_ transcript, it seems likely that the promiscuous GPCRs couple through Gα_s_, as a consequence, these GPCRs are generally included in discussions of Gα_s_ signalling (**Fig 1**).

**Prostaglandin I**_**2**_ **receptor transcript** *(PTGIR)* is the second most abundant GPCR transcript in the pigmented neurons. It codes for the prostacyclin receptor, although it is capable of binding with high affinity to prostacyclin (PGI_2_) as well as prostaglandin E_1_ [30,31] and PGE_2_, although with lower affinity [32].

Prostaglandin synthesis begins with the release of arachidonic acid from cell membrane phospholipids by phospholipase A_2_ (particularly *PLA2G4A* but also *PLA2G4C* [33]), which is then converted by cyclooxygenase I or II (*PTGS1 and PTGS2, respectively*) into prostaglandin H_2_ prior to the production of prostaglandins via specific synthases. In pigmented neurons, *PLA2G4C* and *PTGS1* transcripts are modestly expressed, indicating some capacity to make prostaglandins from endogenous phospholipids, but these low transcript expression levels may also be consistent with the idea that neurons take up arachidonic acid, released by astrocytes, from which they produce prostaglandins [34]. The pigmented neurons show an abundance of prostaglandin E_3_ synthase (*PTGES3*), prostaglandin E_2_ synthase (*PTGES2*) and prostaglandin D_2_ synthase (*PTGDS*) (**Fig 1**), but very little prostaglandin I_2_ synthase (*PTGIS*, **S2 Table**) indicating a capacity to produce prostaglandin mediators (such as prostaglandins E_1/2_, D_2_ as well as J_2_, but not I_2_) which are presumably available for local paracrine signalling and autocrine feedback. There are, however, some differences in substrate utilization for the prostaglandin synthases. Thus, prostaglandin E_2_ is synthesized from arachidonic acid (subject to the activity of phospholipase A_2_ family members). In contrast, prostaglandin E_1_ is synthesized using dihomo-γ-linolenic acid as a precursor, which is in turn derived from dietary linoleic acid via δ5 and δ6 desaturases (*FADS1* and *FADS2*, respectively) and the ELOVL fatty acid elongases 2 and 5 (*ELOVL2* and *ELOVL5*), see [35]. Prostaglandin E_3_ synthase is a cytosolic enzyme capable of producing prostaglandin E_1/2_ while prostaglandin E_2_ synthase is a membrane bound, glutathione-binding prostaglandin synthase that can also play a role in the degradation of the prostaglandin E_2_ precursor, prostaglandin H [36]. That these enzymes do not change in pigmented neurons taken from individuals with PD indicates an unaltered capacity, at least at the transcriptomic level, to produce three main prostaglandin mediators: PGD_2_, PGE_2_ and PGE_1_.

Similar to the pigmented neurons, *PTGDS* is the predominant prostaglandin synthase transcript present in the nigral volume. Although *PTGES3* is also abundantly expressed (and upregulated), the membrane bound, glutathione-dependent synthase, *PTGES2* is far more modestly expressed and significantly lower in PD (**Fig 1**). These data indicate that the transcript for a robust program of prostaglandin D_2_, E_1_ and E_2_ production is still available within the substantia nigra of individuals with PD. However, a significant decrease in *PTGES2* within the nigral volume (likely due to the loss of pigmented neurons) and an increase in *PTGES3* might hint at substantia nigra volume changes in prostaglandin production, which can be seen in toxin models of PD [37]. The increase in *PTGES3* protein (also known as p23) is particularly interesting since it is also a co-chaperone for heat shock protein 90 (HSP90) [38] and is upregulated in numerous cancers (see [39]). Thus, the upregulated nigral *PTGES3* may be more to do with binding incorrectly folded proteins than prostaglandin synthesis.

It is interesting to note that transcript for 5-hydroxyprostaglandin dehydrogenase (*HPGD*), which produces the enzyme responsible for the catabolism of prostaglandins, lipoxins and resolvins (generated from Ω-3 fatty acids by *ALOX5/15*) [40–42] is modestly expressed in pigmented neurons, but barely evident in the nigral volume (**Fig 1**) indicating that it may be largely restricted to neurons.

**Adhesion G Protein-Coupled Receptor L1** (*ADGRL1* transcript), is a member of the latrophilin subfamily of adhesion GPCRs. This family interacts with both matrix components and soluble signals, contributing to CNS development [43] and synapse maintenance/formation/function [44]. *ADGRL1* is alternatively spliced to produce variants of the latrophilin-1 receptor that modulate preference for adenylate cyclase activity [45]. In mature neurons, latrophilins (including ADGRL1) are found post-synaptically where they bind to a number of pre-synaptic proteins including teneurin transmembrane protein 2 (*TENM2*), α-neurexins (*NRXN1–3*), contactin-6 (*CNTN6*, for *cis*- rather than *trans*-binding) and possibly fibronectin leucine-rich transmembrane proteins (*FLRT1–3*), see [46]. While *ADGRL1* expression does not change in pigmented neurons, nigral volume expression of two of its ligands, *NRXN1* and *NRXN3* [47] are significantly lower in PD (**Fig 1**). While the nigral volume shows reductions in these neurexin isoform transcripts, other markers of synapse formation do not change in PD. Thus, pre-synaptic synaptophysin (*SYP*) and (glutamatergic) post-synaptic density protein 95 (*DLG4*) proteins [48] are unaltered within the nigral volume (**S2 Table**). Whether this indicates that the loss of nigral *NRXN1* and *NRXN3* is likely in response to the loss of pigmented neurons should be determined empirically.

In contrast to *NRXN1* and *NRXN3*, *NRXN2* expression does not change in the nigral volume indicating a more widespread expression of this transcript within the cells of the substantia nigra, rather than just the pigmented neurons. Within the caudate nucleus and putamen *NRXN1* transcript increases, which seems likely as a mechanism for increasing support for the remaining synapses, although why it is only restricted to *NRXN1* is unclear.

**G-protein coupled receptor 26** (*GPR26*) is a constitutively active orphan GPCR abundantly and widely expressed in the CNS, including the substantia nigra [49]. While expressed in relative abundance in the pigmented neurons it does show, in contrast to many of the other orphan receptors, a significant decrease in expression within the nigral volume (**S2 Table**), perhaps indicating some level of specificity for expression within pigmented neuron population. At present its activating ligand(s) is/are unclear.

**Frizzled 3 receptors** (*FZD3*) are located on soma, dendrites, axons and synapses and are commonly associated with WNT/PCP signalling [50] although it has been shown to respond to Wnt5a to promote adenylate cyclase activity [51]. The generally weak expression of *WNT5A* transcript in the nigral volume along with relatively low levels of *FZD3* transcript may indicate that WNT-stimulated adenylate cyclase activity represents a reasonably minor fraction of total adenylate cyclase activity (**Fig 1**). Note that *FZD3* is much more highly expressed within the caudate nucleus and putamen (**S2 Table**). While there is an abundance of roof plate specific spondin-2 (*RSPO2*) as well as dickkopf-3 (*DKK3*) transcripts within pigmented neurons, current evidence indicates that it is unlikely that they activate FZD3-Gα_S_ signalling. Their ability to regulate WNT/PCP pathways is discussed below.

Generally, the current data indicates some potential for changes in Gα_S_ coupled GPCR activation within the pigmented neurons, at least with respect to the most abundant Gα_s_-coupled GPCR transcripts. Overall, it appears that there may be reduced neurexin (*NRXN3/1)* transcripts available for neuron-neuron signalling through *ADGRL1*, as well as altered prostaglandin E-series production within the volume of the nigra. That there are changes in signal-related transcripts, might indicate a potential for changes in signal transduction within the pigmented neurons. Thus Gα_s_ signal transduction-related and allied transcripts were assessed in pigmented neurons.

#### 3.2.1. Gα_s_ Transduction processes.

Traditional pathways of Gα_s_ coupled GPCR receptor activation require stimulation of adenylate cyclase isoforms. It’s interesting to note that while multiple adenylate cyclases are present in pigmented neurons, they are not particularly abundant (**Fig 1**, **S2 Table**) and relatively restricted to three isoforms: 5, 3 and 2 (*ADCY5*, *ADCY3* and *ADCY2*). Once expressed these adenylate cyclase isoforms can all be activated directly by Gα_s._ Beyond changes in transcript expression, there are multiple intrinsic levels of interaction that might alter cellular adenylate cyclase protein activity. For example, adenylate cyclase 5 is subject to negative regulation by protein kinase A, regulator of G-protein signalling 2, and likely protein associated with myc (*MYCBP2*); but positive regulation by protein kinase Cα/ζ isoforms (see [52]). The calcium-calmodulin complex can also activate the group I adenylate cyclases (ADCY1/3/8); noting that adenylate cyclase 1 is similarly activated at resting levels of cellular calcium [53]. ADCY2 is additionally activated by βγ-subunits [54]. While there is no evidence of change in transcripts for any of these known adenylate cyclase modulatory units, the sum of their protein contributions to the regulation of enzyme activity in pigmented neurons is unknown.

Once produced by adenylate cyclase, cAMP activates both protein kinase A and EPAC. Many PKA catalytic (*PRKAC*) and regulatory (*PRKAR*) isoform transcripts are robustly expressed in pigmented neurons (**Fig 1**). The kinase holoenzyme is a scaffold-bound tetramer strictly composed of two regulatory and two catalytic subunits. Upon binding cAMP, the catalytic subunits are conformationally liberated or released, and the enzyme is free to phosphorylate substrates [55]. It seems that regulation is a vital aspect of PKA function since there are around twice as many regulatory than catalytic subunit transcripts (**Fig 1**). Regardless, once activated, PKA phosphorylates a variety of substrates to either increase or decrease activity. The list of substrates includes multiple kinases and both potassium and calcium channels (see [56]). With respect to those systems showing appreciable levels of transcript expression, PKA can, for example activate C-Src Kinase (*CSK* [57]) to, in turn, activate or inhibit activity of the Src family member, FYN (*FYN*) [58,59], possibly indicating a context or isoform-dependent, or more likely scaffold/recruitment effect (see [58]). PKA can also inhibit the protein kinase, RAF1 (*RAF1*) to inhibit ERK signalling [60] as well as inhibit/stimulate mTORC1 activity (see [61]). While *RAF1* is modestly expressed, pigmented neurons show little expression of the mTORC1 complex member, Regulatory Associated Protein Of MTOR Complex 1 (*RPTOR*), **Fig 1**. Perhaps the most critical pro-survival relationship is between PKA and glycogen synthase kinase (*GSK3A* and *GSK3B*). In vitro, PKA (and AKT) phosphorylate GSK3β (Ser9) to inhibit GSK activity and promote neuron survival [62], an effect mimicked by calmodulin kinase-2 (*CAMK2*), which also exerts a similar effect upon (Ser21) GSK3α [63]. The pigmented neurons show substantial expression of both *GSK3A* and *GSK3B* (as well as three of the four *CAMK2* isoforms, discussed below). Concurrently, mitochondrially anchored PKA can phosphorylate and inhibit the activity of the pro-apoptotic mediator, BAD (*BAD*) to facilitate binding to 14-3-3 protein family members, preventing inhibition of the activity of the pro-survival BCL2 family members (*BCL2L1* and *BCL2L2*) [64]. Note that there is very abundant *BAD* and also 14-3-3 family member transcripts (*YWHAG/B/H/E/Z/Q*) as well as PKA scaffold transcripts expressed in the pigmented neurons (**Fig 1**).

PKA can also directly regulate the activity of several robustly expressed ion channels within the pigmented neurons. These multimeric ion channels include Shal-related, voltage gated Kv4.3 (*KCND3* is the most abundant transcript); multiple G-protein-activated inward rectifier channels (GIRKs, *KCNJ6* is the most abundant); the voltage gated Kv7.3 (*KCNQ3* and *KCNQ2* are equally abundant) and the IP_3_ receptor (*ITPR1*) (see [56]). The relative abundance of these transcripts provides a clue to likely membrane compositions of these channels. For example, the comparatively high level of *KCND3*, indicates the likely formation of KCND3 predominant Kv4.3 channel tetramers since the next highest Kv4 channel transcript, *KCND2* is only 15% of its abundance (**Fig 1**). This channel is found in both the soma and somatodendritic regions where it rapidly activates and inactivates in response to depolarization; it is commonly found in a complex with calcium-binding K^+^-Channel-Interacting-Proteins (*KCNIP*s) and dipeptidyl-peptidase-like-proteins (*DPP6* and *DPP10*) which modulate channel activity (see [65]). Of these, *DPP6* transcript is by far the most abundant (**Fig 1**). Phosphorylation of Kv4 channels by PKA reduces cell surface expression thereby reducing current [66].

GIRKS are inward rectifying K^+^ channels, commonly with multiple splicing variants, that mediate post-synaptic inhibitory effects via binding up to four Gβγ-protein subunits along with PIP_2_ (see [67]). These channels exhibit low level tonic activity and their inhibition results in cellular depolarization [67]. Given the abundance of *KCNJ6* over *KCNJ9* it seems that the majority of these channels are likely to be GIRK2 (*KCNJ6*) homo- and GIRK2/3 (*KCNJ6/9*) hetero-tetramers which may be subject to positive modulation by both PKA-dependent phosphorylation as well as Gβγ subunit binding [68]. The pigmented neurons also modestly express Kv7.3 channel transcripts (*KCNQ* isoforms) coding for channels that maintain a negative resting membrane potential to reduce neuron excitability, an effect which is also enhanced by PKA-dependent phosphorylation [69]. Thus any change in PKA activity is likely to modulate neuron excitability. Whether reductions in neurexin expression or changes to prostaglandin synthesis impacts activity in the pigmented neurons remains to be seen.

The pigmented neurons also contain multiple ligand gated Cl^-^ channel subunits, notably GABA (predominantly *GABRG2*, *GABRB1/2/3*, *GABRA1* and *GABRA4*, discussed later) and glycine (*GLRA3* and *GLRB)* subunit transcripts (**S2 Table**). Reflecting transcript expression, the majority of the glycine receptors are likely α3β pentamers (**Fig 1**) which can be subject to inhibitory PKA phosphorylation (Ser346) [70,71]. The pigmented neurons also express voltage gated calcium channel transcripts representing the P/Q-type (*Ca*_*v*_2.1, *CACNA1A*), N-type (*Ca*_*v*_2.2, *CACNA1B*), R-type (*Ca*_*v*_2.3, *CACNA1E*) and T-type (*Ca*_*v*_3.1, *CACNA1G*), **Fig 1**. Generally, PKA potentiates the activity of voltage gated calcium channels [72–74], discussed below.

Lastly, cAMP also activates Exchange Protein Directly Activated by cAMP 1 (EPAC1) and 2 (EPAC2), which are Rap guanine nucleotide exchange factors-3 (*RAPGEF3*) and −4 (*RAPGEF4*), respectively. The protein for the most abundant transcript, *RAPGEF4,* likely exists as the CNS-enriched EPAC2 splice variant(s) which acts as a guanine nucleotide exchange factor (GEF) for the monomeric GTPases; RAP1A, RAP1B and RAP2A (the most abundant RAP, **S2 Table**) [75]. RAPGEF4 activity is associated with the inhibition of GIRKs [76] and has been found to promote dendritic spine shrinkage and depress excitatory transmission [77] (**Fig 1**). In contrast, the weakly expressed RAPGEF5 is a cAMP-independent activator of RAP1/2 [78]. Once activated RAPs play a number of roles with the cells including the regulation of cell-cell contact, adhesion and actin remodelling (via RHO/RAC) [78] and the activation of Raf signalling [79] possibly by increasing AKT stability through binding SNAP25 [80]. RAP family members are also important regulators of intracellular calcium (at least for RAP1) [81] and the activation of the kinase effectors TRAF2 and NCK Interacting Kinase (*TNIK*) and Mitogen-Activated Protein Kinase Kinase Kinase Kinase 4 (*MAP4K4*) (see [82]). While *TNIK* (and the related *MINK1*) and *MAP4K4* are weakly expressed, another RAP2A effector, RUN Domain Containing 3A (*RUNDC3A*) shows highly abundant transcript expression in pigmented neurons. Opposing RAPGEF activity, the pigmented neurons express transcript for two RAP GTPase activating domain containing proteins, Signal Induced Proliferation Associated 1 Like 2 & 1 (*SIPA1L2/1*) as well as the robustly expressed transcript *RAP1GAP* (**Fig 1**).

Ultimately it is impossible to predict exactly how PD impacts adenylate cyclase activity in pigmented neurons. Analysis of signal transduction transcripts indicates little to no change in the ability to produce or metabolize cAMP within the pigmented neurons. Thus, it seems likely that, if cAMP production is affected in PD, it is likely via changes in signal input to Gɑ_s_ coupled GPCRs. Whether the most abundant Gα_s_ coupled receptor transcript, the prostaglandin I_2_ receptor (*PTGIR*), is likely to be impacted by PD remains unclear. The prostaglandin I_2_ receptor has been shown to regulate transforming growth factor-β and bone morphogenetic protein receptor type 2 BMPRII signalling (see [83]), effects that may be linked to Gα_s_ activity. In addition this receptor has been linked, more broadly to neuron survival in the CNS [37]. Based on nigral prostaglandin synthesis transcript expression levels, likely impacts on prostaglandin I_2_ receptor signalling will be dependent upon the ability of prostaglandins D_2_ and E_1/2_ to bind to and potentially activate this receptor. In addition, while the loss of neurexin expression may contribute to altered ADGRL1-driven Gα_s_ stimulation, this depends upon which splice variants are present in pigmented neurons. Confounding any interpretation of PD-related changes in Gα_s_ signalling in the pigmented neurons are the unknown ligands and contributions of the orphan receptors, GPR162, GPRC5B, GPR158 and GPR161 (**S2 Table**).

While not all directly related to the regulation of PKA phosphorylation dependent activity, **Fig 1** shows a list of serine-threonine phosphatases. Note the high expression of protein phosphatase 1 (*PPP1*) and protein phosphatase 2 (*PPP2*) family transcripts whose proteins are capable of dephosphorylating phospho-serine/threonine residues on a variety of substrates, including AKT [84] and the Shal-related K + -DPP6 complex [85], and which can be indirectly regulated by cAMP/PKA (see [84]).

### Gα_o_ coupled GPCR signalling

The Gα_i/o_ family consists of multiple family members, including Gα_i1_ (*GNAI1*), Gα_i2_ (*GNAI2*), Gα_i3_ (*GNAI3*), Gα_o_ (*GNAO1*), Gα_t_ (*GNAT1, 2* and *3*), and Gα_z_ (*GNAZ*), which show varying capabilities in repressing adenylate cyclase. Notably, the most abundant family member transcript (*GNAO1*) represents a protein (Gα_o_) that appears relatively ineffective at repressing adenylate cyclase activity (see [86]). In contrast to the relative stability of GPCR-Gα_s_ -dependent receptor activation and signal transduction, Gα_i/o_ family member GPCR signalling shows more widespread changes, particularly within the nigral volume. Thus, there are multiple transcripts for GPCRs with Gα_i/o_ family primary coupling, including the highly expressed dopamine D_2_ receptor (*DRD2*), the abundantly expressed GABA_B1_ (*GABBR1*) and GABA_B2_ (*GABBR2*) heterodimer receptor subunits as well as the significantly downregulated somatostatin receptor-1 transcript (*SSTR1*) (**S2 Table**). Of these receptors the DRD_2_ and GABA heterodimer are likely to be associated with Gα_o_ signalling.

**Fig 2:**
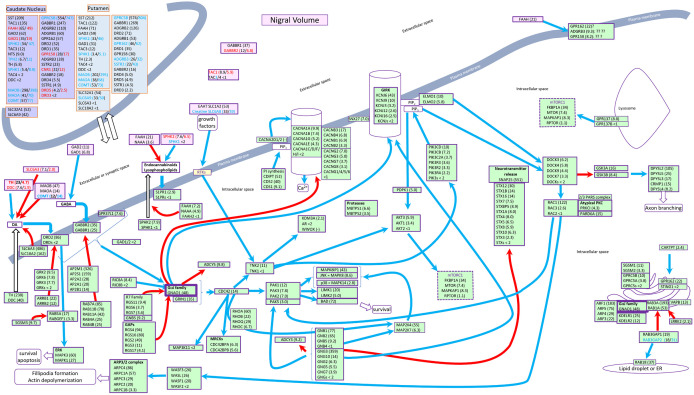
Speculative Gα_o_ family signalling pathways in pigmented neurons. The image includes gene symbols for transcripts for ligands presumed present in the nigral volume and/or synaptic spaces (pink boxes), putamen or caudate nucleus (mauve and blue boxes, respectively) and the pigmented neurons (green boxes). The numbers in the brackets represent the transcript per million (TPM) from control tissue/cells. If the differential expression analysis (DEA) of the data set shows a significant change transcript associated with Parkinson’s disease, then the gene name is coloured red for a decrease and blue for an increase. The significant change in expression (control TPM adjusted by DEA value) is represented by the number after the forward slash (/). The white boxes (for example, the one containing PIP_2_) generally indicate a process or substance produced, introduced into or activated within the intra- or extra-cellular space. The red arrows indicate an inhibitory action while the blue arrows indicate a stimulatory action (sometimes blue and red arrows connect the same pathways to indicate context dependent signalling). The white arrows indicate a potential for substance movement across membranes. When boxes are connected it indicates a likely protein binding partner (atypical PKC (*PRKCI*) and *PARD6A*, for example). Some transcripts, for example some of the syntaxins (*STX19* and *STX11*) are weakly expressed and listed as other STXs < 2, indicating that remaining family member transcripts are expressed at less than 2 TPM (**S2 Table** shows all transcript values). This is a stylized map of activity it is not intended to define subcellular or the extracellular locations of transcript derived proteins with any degree of accuracy. See text for details of major pathway interactions and definitions.

***DRD2*** is the most highly expressed transcript present in pigmented neurons. The D_2_ dopamine receptor it codes for is predominantly coupled through Gα_i/o_, but it can recruit AKT through β-arrestin [25] to regulate a vast array of intracellular signalling cascades. DRD_2_ can exist as long or short isoforms with evidence indicating that the short isoform is likely more abundant on pigmented neurons (see [87–90]). However, recent evidence indicates that the long form of D_2_ receptor preferentially activates Gα_o/z_ rather than Gα_i_ [91]. That, within the pigmented neurons, *GNAO1* is the most abundant transcript of the Gα_i/o_ family (**Fig 2** and **S2 Table**) is not inconsistent with the two ideas that the major mode of neurotransmission for nigrostriatal dopaminergic neurons is short distance extrasynaptic volume transmission (see [92]) and that dopaminergic neurons likely provide feedback inhibition to adjacent dopaminergic neurons via D_2_ dopamine receptors (see [93]). Thus, it seems likely that within the substantia nigra the D_2_ dopamine receptor’s main function is to elicit intracellular signalling via Gα_o_ and or Gα_i2_ G-proteins associated with β_1_γ_3_ or β_2_γ_3_ subunits. It therefore seems likely that the significant losses of *TH* and *DDC* from the nigral volume will negatively impact local DRD_2_-directed intracellular signalling.

***GABBR1*** and ***GABBR2*** are highly expressed transcripts within the pigmented neuron population. This GPCR functions as a heterodimer where the GABA_B1_ (*GABBR1*) receptor is essential for ligand binding and the GABA_B2_ (*GABBR2*) is responsible for G-protein coupling (see [94]). There are multiple isoforms of GABA_1_, dependent upon differential transcription or splicing and these impact likely locations of the functional protein. Thus, the GABA_B1a_ receptor isoform, usually associated with pre-synaptic sites, is different to the GABA_B1b_ receptor isoform present in post-synaptic sites (although this isoform association with location is not exclusive, see [95]). Like the D_2_ dopamine receptor, GABA_B_ receptors (as a heterodimer) have been suggested to activate Gα_o_ and either Gα_i1_ and/or Gα_i2_ receptors (see [96]), most likely Gα_i2_ since *GNAI2* is ~ 10x more abundant than *GNAI1* (**S2 Table**) within pigmented neurons.

The high expression of both *DRD2, GABBR1/2* and *GNAOI* transcripts indicates that the activation of Gα_o_ signal transduction is a key component of pigmented neuron function.

#### 3.3.1. Transduction processes.

Gα_o_ is particularly interesting within the context of signal transduction and disease. Mutations of this gene are associated with epilepsy, developmental delay and movement disorders [97,98]. Besides inhibition of adenylate cyclases, Gα_o_ has been suggested to interact with several signalling systems. It can, for example, inhibit N-, and P/Q type (Ca_v_2.2 and 2.1) channel activity, either alone or via the action of liberated βγ subunits (see [99]), as well as stimulate G protein-coupled inward rectifying potassium (GIRK) channel opening [98] (**Fig 2**). Alongside these actions Gα_o_ can bind to G protein-regulated inducer of neurite outgrowth 1 (*GPRIN1*) to activate the Rho GTPase family member cell division control protein 42 (*CDC42*) [100]. CDC42 is central to the regulation of numerous cellular processes including activation of Tyrosine Kinase Non Receptor 2 (also known as activated Cdc42-associated kinase 1 (*TNK2*), a multifunction protein with kinase activity capable of interacting with and regulating the activity of a number of proteins, including the androgen receptor (*AR*), Lysine Demethylase 3A (*KDM3A*), WW Domain Containing Oxidoreductase (*WWOX*) and Akt Serine/Threonine Kinase family members (*AKT1/2/3*) [101]. It can also interact with and stabilize PI3 kinase regulatory subunits [102]. Of these listed TNK2 protein interacting proteins, only the PI3K members are appreciably expressed, albeit moderately (**Fig 2**, see also **S2 Table**). In addition to AKT signalling, PI3K can also stimulate the activity of another monomeric G-protein, rac1 (*RAC1* transcript is very abundantly expressed) via a PIP_3_ mediated interaction of Dedicator Of Cytokinesis (*DOCK*) protein family members with Elmo1/2 (engulfment and cell motility 1 and 2: *ELMO1/2*) family members [103]. CDC42 is also linked to the activation of P21 (RAC1) Activated Kinase (*PAK1/2/3*) family members which activate p38 (*MAPK14*) and JNK (*MAPK8*) and LIM1 kinases (*LIMK1/2*) while also directly promoting survival through phosphorylation of BAD [104]. Other effectors also regulate these pathways. Thus, βγ subunits have been shown to activate p38 and JNK kinases (**Fig 2**), albeit via different monomeric G-proteins [105]. The very robust expression of Mitogen-Activated Protein Kinase 8 Interacting Protein 1 (*MAPK8IP1*), a scaffold protein that selectively mediates JNK signalling [106] and autophagosome motility [107] seems likely to indicate potential for JNK signalling as directed by intracellular signalling.

Interestingly, DOCK3 protein binds to and inactivates glycogen synthase kinase-3β (GSK-3β) decreasing phosphorylation of the collapsin response mediator protein-2 family member 2 (*DPYSL2*), which promotes axon branching and microtubule assembly [108]. Concurrently, RAC1 modulates actin dynamics via the Wiskott-Aldrich syndrome family of proteins (WAVE and WASP), which signal through the actin-related protein-2/3 (ARP2/3) complex [109]. This complex orchestrates actin polymerization within both the soma and the nucleus, where it is known to facilitate the nuclear repair of damaged chromatin [110]. The ability of Gα_o_ proteins to regulate actin polymerization is interesting given that PD is associated with a loss of synaptic terminals in the substantia nigra (see [111]) and, perhaps more broadly, that *GNAO1* mutations are linked to movement disorders [98].

Of course, GPCR signal transduction is impacted by other factors beyond the availability of signal.

### Regulators of G-protein coupled receptor signalling

Following agonist binding to GPCRs, G-protein heterotrimers dissociate to elicit effects and reassemble ready for the next signalling cycle. To speed up this process, GTPase activating proteins (GAPs) stimulate hydrolysis of bound GTP to inactivate the Gα-subunits and promote the re-formation of the G-protein heterotrimer. Most commonly these GTPases fall into the regulator of G-protein signalling (RGS) family. Of the 20 RGS members, three transcripts are very highly expressed in pigmented neurons: *RGS4*, *RGS16* and *RGS2* (**Fig 2**). Of these proteins, RGS4 shows some preference for Gα_i/o_ over Gα_q_, RGS16 appears equally effective at Gα_i/o_ and Gα_q_, while RGS2 is selective for Gα_q_ [112]. That much of the GAP transcript activity present in these neurons appears directed at minimising Gα_i/o_ family signalling seems consistent with the idea that this G-protein family, and in particular GPCR directed Gα_o_ signalling, is critical for normal cell function.

An additional level of control of GPCR signalling occurs at the plasma membrane when stimulated GPCRs are phosphorylated by G-protein receptor kinases (*GRK2*, *GRK3* and *GRK6* transcripts are the most abundant) to enable β-arrestin (*ARRB1/2*) recruitment and internalization (**Fig 2**). Functionally, GRK2 and GRK6 have been shown to respectively regulate pre- and post-synaptic D_2_ dopamine receptors [113], establishing GRK2 as a key modulator of somatodendritic autoreceptor feedback within these dopaminergic neurons. Once internalized, the D_2_ dopamine receptor is either degraded, recycled or continues G-protein independent signalling. The long form of the D_2_ dopamine receptor has, in a complex with RABGEF1 (*RABGEF1*) and RAB5 (*RAB5*), been shown to drive ERK1/2 (*MAPK3*, *MAPK1*) activation. ERK1 and ERK2 subsequently activate multiple cellular signalling cascades that regulate survival (see [114]).

In contrast with the D_2_ dopamine receptors, the GABA_B_ receptor heterodimer appears to have a slightly different desensitization program where the GABA_B_ receptor is recruited to clathrin-coated pits by the AP2 heterotetramer complex (*AP2A1, AP2A2, AP2M1, AP2S1* and *AP2B1*) prior to internalization and sorting into Rab4, Rab7 or Rab11 associated endosomes (see [115]). While the stoichiometry of the AP2 complex is unknown, it is interesting to note that the *AP2M1* and *AP2S1* subunits are in excess of the other family members. There is also an abundance of *RAB7A*, *RAB11B* and *RAB11A* transcripts suggesting that intracellular trafficking is predominantly RAB7A, RAB11B and RAB11A directed. At present there is, however, little evidence to suggest that internalized GABA_B_ receptors continue to signal as do D_2_ dopamine receptors. Regardless of GABA desensitization and trafficking programs, local GABAergic interneuron GABA production seems likely to remain unchanged since nigral volume *GAD1* and *GAD2* expression do not change in PD (**Fig 2**). There is, however, a reduction in caudate nucleus *GAD1* transcript associated with PD. This change is one of the few differences between caudate nucleus and putamen gene expression in PD tissue. Its biological significance is unclear.

Generally, these data support a prominent role for dopamine D_2_ receptor-driven Gα_o_ involvement in the modulation of ion channels, ERK and BAD activity (pro-survival), cytoskeletal/dendritic modelling (via WASP and ARP2/3 family complexes), repair of damaged DNA and pro-stress pathways (JNK). While presumptions of normal pigmented neuron dopamine production are supported by the lack of change of both *TH* and *DDC* transcripts (noting that tyrosine hydroxylase protein activity is highly regulated [116]), the loss of nigral volume *TH* and *DDC* (**Fig 2**) is consistent with a deficit in local dopamine feedback. This, in turn, seems likely to impact the ability of the dopamine D_2_ receptor to activate Gα_o_ and βγ subunit driven pro-survival mechanisms.

### Gα_i_ and Gα_z_ coupled GPCR signalling

**Fig 3:**
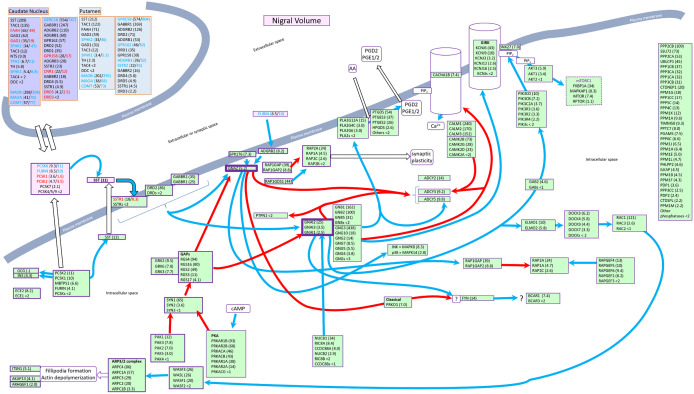
Speculative Gα_i/z_ family signalling pathways in pigmented neurons. The image includes gene symbols for transcripts for ligands presumed present in the nigral volume and/or synaptic spaces (pink boxes), putamen or caudate nucleus (mauve and blue boxes, respectively) and the pigmented neurons (green boxes). The numbers in the brackets represent the transcript per million (TPM) from control tissue/cells. If the differential expression analysis (DEA) of the data set shows a significant change transcript associated with Parkinson’s disease, then the gene name is coloured red for a decrease and blue for an increase. A significant change in expression is represented by the number after the forward slash (/) which is the TPM x DEA value. The white boxes (for example, the one containing PIP_2_) generally indicate a process or substance produced, introduced into or activated within the extra- or intracellular space. The red arrows indicate an inhibitory action while the blue arrows indicate a stimulatory action (sometimes blue and red arrows connect the same pathways to indicate context dependent signalling). The white arrows indicate a potential for substance movement across membranes. When boxes are connected it indicates a likely binding partner (*CALM* and *CAMK2*, for example). Some transcripts, for example some of the phosphatases, are weakly expressed and listed as other phosphatases <2, indicating that remaining family member transcripts are expressed at less than 2 TPM (**S2 Table** shows all transcript values). This is a stylized map of activity it is not intended to define subcellular or the extracellular locations of transcript derived proteins with any degree of accuracy. See text for details of major pathway interactions and definitions.

There are three Gα_i_ subtypes, Gα_i1_ (*GNAI1*), Gα_i2_ (*GNAI2*) and Gα_i3_ (*GNAI3*). Of these, *GNAI2* transcript is by far the most abundant in the pigmented neurons, with modest levels of *GNAI1*, *GNAI3* and also *GNAZ* expressed (**Fig 3**). Gα_i_ is usually associated with the inhibition of adenylate cyclase activity (see [117]) but the final effects upon adenylate cyclase activity are more nuanced. Thus, of the three most commonly expressed ADCY transcripts expressed in pigmented neurons; *ADCY2*, *ADCY5* and *ADCY3.* Adenylate cyclase-2 is activated by Gβγ, and both PKC- and Raf-phosphorylation. Adenylate cyclase-3 is activated by PKC- and the Ca^2+^-calmodulin complex, but inhibited by RGS2, Gβγ and CAMK2-phosphorylation. Adenylate cyclase-5 is directly inhibited by Gα_i_ subunits and PKA phosphorylation, while being positively modulated by PKC and Raf phosphorylation, and regulated in a context-dependent manner by Gβγ subunits [118], **Fig 3**.

There are several GPCR transcripts for receptors that couple through Gα_i/o_ family members including the abundantly expressed GABA_B_ and D_2_ dopamine receptors, discussed above, but also the somatostatin (*SSTR1*) and orphan Gpr176 (*GPR176*) receptors which seem more selective in Gα_i/o_ family member binding.

***SSTR1*** is relatively highly expressed in pigmented neurons, however, this is the only GPCR transcript to show a significant reduction (~50%) associated with PD (**Fig 3**). This is perhaps consistent with an early report that somatostatin could reduce dopamine release from brain tissue [119], suggesting this as a possible mechanism to increase dopamine release in the face of reducing dopaminergic neuron input to local and striatal tissues. Later work identified that somatostatin receptors (at least in rat brains) preferentially coupled through Gα_i_, but not Gα_o_ G-proteins [120]. Other data indicates that somatostatin receptors appear particularly efficient in coupling through Gα_z_ [121]. At the functional level, somatostatin receptors can form heterodimers with D_2_ dopamine receptors [122], although the evidence for SST_1_ receptor - D_2_ dopamine receptor heterodimers is not as clear cut as it is for other SST receptor subtypes. Regardless, the pigmented neurons seem likely to possess somatostatin receptors that inhibit neurotransmitter release through a reduction of adenylate cyclase activity. While the receptor transcript is clearly expressed within the pigmented neurons, so too is the somatostatin (*SST*) transcript, as well as the major proprotein convertase; Proprotein Convertase Subtilisin/Kexin Type 2, *PCSK2* [123], indicating a potential for autocrine feedback. While *SST* and *PCSK2* do not change in pigmented neurons, *PCSK2* is significantly reduced in the nigral volume perhaps indicating reduced ability to produce SST. In contrast to the nigral volume, the putamen and caudate nucleus contain substantially more *SST* (significantly downregulated) and *SSTR2* (significantly upregulated) (**Fig 3** and **S2 Table**).

***GPR176*** codes for an orphan GPCR that has been shown to couple through Gα_z_ (*GNAZ*), a pertussis toxin-insensitive G-protein subunit with low intrinsic GTPase activity, to inhibit adenylate cyclase and regulate the circadian clock (see [124]). Activated Gα_z_ is also able to recruit Rap1GAP (*RAP1GAP*) from the cytosol to the membrane [125] where it may inactivate the Ras-Related Protein GTPases Rap-1A and Rap-2A (*RAP1A* and *RAP2A*) [126]. Interestingly, while *RAP1GAP* transcript is highly expressed, so too is the Rap1 GTPase-GDP Dissociation Stimulator 1 (*RAP1GDS1*), a guanine nucleotide exchange factor [126] (**Fig 3**). The high expression of Raps and Rap regulators within the pigmented neurons is consistent with them having a role in the regulation of synaptic plasticity [127].

**Adhesion G Protein-Coupled Receptor B2 (*ADGRB2*)** is a member of the adhesion receptor family. This GPCR (formerly known as BAI2), like GPR176, preferentially couples through Gα_z_ [128], but is regulated and processed through proteolytic processing via furin (*FURIN*), a widely expressed calcium dependent membrane bound endoprotease that can undergo shedding into the extracellular space, and potentially other enzymes (see [129]). *FURIN*is significantly upregulated within the PD nigral volume but more modestly expressed (and unchanged) within pigmented neurons (**Fig 3**).

#### 3.5.1. Transduction Processes: Gα_i_.

Typically, neuronal Gα_i_ activation is seen as a canonical mechanism to reduce neurotransmitter release. These G-proteins will also, however, contribute to a range of other cellular activities including the suppression of tyrosine phosphatase 1B (*PTPN1*) [130], the activation (along with Gα_s_) of Src family tyrosine kinases [131]; note that *FYN* transcript is the most robustly expressed member of the Src family (**S2 Table**). In addition Gα_i_ has been linked to the activation of the PI3 kinase-Akt-mTORC1 pathway positively regulating cell survival/proliferation in response to growth factor stimulation via growth-factor receptor binding 2 [Grb2]-associated binding protein 1 (*GAB1*) (see [132]), **Fig 3**. In addition, Gα_i2_ can promote the phosphorylation of p130Cas (*BCAR1*) by Src family kinases [133] as well as stimulate the translocation of Engulfment And Cell Motility 1 protein (*ELMO1*) to the plasma membrane which enables binding to Dock180 (*DOCK1*) to activate Rac signalling leading to the modification of cytoskeletal structure [134], **Fig 3**.

There are also additional mechanisms that potentiate Gα_i_ -protein activation, these include the G-protein interactors: Coiled-Coil and HOOK Domain Protein 88 members (*CCDC88A/B/C*), RIC8 Guanine Nucleotide Exchange Factors (*RIC8A/B*) and nucleobindin (*NUCB1/2*) proteins [135]. Of these**,** *NUCB1* is robustly expressed (**Fig 3**) indicating a role for this G-protein regulator in directing Gα_i_ signalling.

#### 3.5.2. Transduction Processes: Gα_z_.

Gα_z_ is one of the Gα_i/o_ family members and is commonly included as a member capable of inhibiting adenylate cyclase activity (in a pertussis toxin-insensitive way). However, like Gα_o_, Gα_z_ signalling interacts with a more complex set of downstream effectors than its inclusion in the Gα_i/o_ family of adenylate cyclase inhibitors might indicate. One of the earliest reports of non-adenylate cyclase coupling through Gα_z_ was from Meng et al., who demonstrated that Gα_z_ bound to the RAP1 GTPase Activating Protein (*RAP1GAP*), to block Gα_z_ GTP hydrolysis promoting Ras-Related Protein Rap-1A (*RAP1A*) activity [136]. In pigmented neurons, *RAP2A* is the most abundantly expressed small GTPase of the RAP subfamily (**Fig 3**), and while similar interactions are yet to be determined empirically, it does highlight the possibility of complex G-protein subunit signalling, beyond the simple inhibition of adenylate cyclase activity.

Gα_z_ also interacts with additional transduction machinery; Tu and co-workers found that a variant of one of three synapsin isoforms (IA) inhibited, in a Gα_z_ specific manner, the GTPase-activating protein (GAP) activity of several RGS proteins (the cells robustly express *RGS4/16/2*). Moreover, they found that this inhibition could be attenuated by phosphorylation of synapsin IA (*SYN1*) by either cyclic AMP-dependent protein kinase (*PRKACA/B* subunits) or p21-activated protein kinase (*PAK*) [137]. Gα_z_ is also thought to interact with Src, in a PKCγ (*PRKCG*)-sensitive way to stabilize Src in its inactive form [138], it is currently unclear whether *FYN,* the most abundant Src family member transcript in pigmented neurons (**Fig 3**), is subject to the same regulation.

Additionally, Gα_z_ G-proteins interact with a subset of receptors (such as somatostatin receptors) to inhibit N-type Ca^2+^ channels (*CACNA1B*) [121] while stimulating heteromeric GIRK channels (such as *KCNJ6*) [121]. It can also inhibit Gα_12/13/q_-dependent Rho GEF-activity [139]. Perhaps of most significance is the finding that Gα_z_ (along with Gα_i1-3_ and Gα_o_) activity links to the stimulation of JNK signalling [140] (**Fig 3**). It has also been reported that Gα_z_ regulates BDNF-induction of axon growth in cortical neurons [141], but the nigral volume, caudate nucleus and putamen possess little BDNF transcript (**S2 Table**).

While *GNAZ* and *GNAI1/2/3* transcripts are not quite as abundant as *GNAO1* there is considerable evidence indicating that Gα_z/i_ family member signalling can modulate ion channels and inhibit PI3 kinase and monomeric G-protein activities. How these activities might impact pigmented neurons, particularly with the significant reduction in *SSTR1* expression, is currently unknown.

### Gα_q/11_ coupled GPCR signal transduction

**Fig 4:**
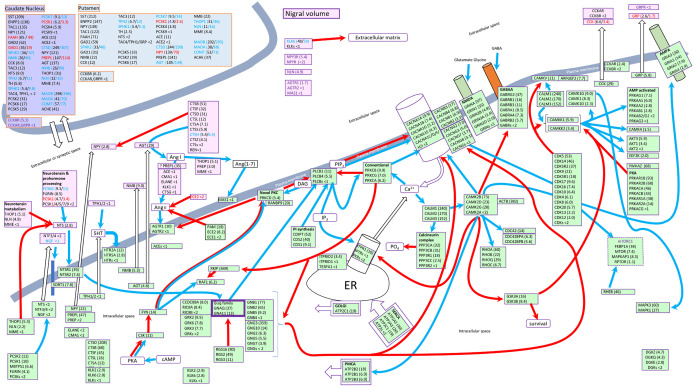
Speculative Gα_q/11_ family signalling pathways in pigmented neurons. The image includes gene symbols for transcripts for ligands presumed present in the nigral volume and/or synaptic spaces (pink boxes), putamen or caudate nucleus input to the substantia nigra (mauve and blue boxes, respectively) and the pigmented neurons (green boxes). The numbers in the brackets represent the transcript per million (TPM) from control tissue/cells. If the differential expression analysis (DEA) of the data set shows a significant change transcript associated with Parkinson’s disease, then the gene name is coloured red for a decrease and blue for an increase. The significant change in expression (control TPM adjusted by DEA value) is represented by the number after the forward slash (/). The white boxes (for example, the one containing PIP_2_) generally indicate a process or substance produced, introduced into or activated within the cellular space. The red arrows indicate an inhibitory action while the blue arrows indicate a stimulatory action (sometimes blue and red arrows connect the same pathways to indicate context dependent signalling). The white arrows indicate a potential for substance movement across membranes. When boxes are connected it indicates a likely binding partner (Gβ and Gγ subunits, for example). Some transcripts, for example some of the G-protein coupled receptor kinases (GRKs), are weakly expressed and listed as other GRKs < 2, indicating that remaining family member transcripts are expressed at less than 2 TPM (**S2 Table** shows all transcript values). This is a stylized map of activity it is not intended to define subcellular or the extracellular locations of transcript derived proteins with any degree of accuracy. See text for details of major pathway interactions and definitions.

Within the pigmented neuron population, the Gα_q/11_ family members Gα_q_ (*GNAQ*) and Gα_11_ (*GNA11*) are clearly present while the remaining family members: *GNA14* and *GNA15*, are weakly expressed (**S2 Table**). The robustly expressed neurotensin receptor (*NTSR1*), serotonin receptor 2A (*HTR2A*) and angiotensin II type 1 receptor (*AGTR1*) are linked to signalling via this G-protein family (**Fig 4**).

**Neurotensin receptor 1 (*NTSR1*)** is the most abundant of the Gα_q/11_-preferring GPCR transcripts. Activation of this signalling pathway leads, either directly or indirectly, to stimulation of RAF1, SRC family members and protein kinase D (*PRKD1/2/3*) related pathways. It can also activate monomeric G-protein (*RAC/RHO/CDC42*) related pathways (see [142]), **Fig 4**. While its transcript is abundantly expressed in its own right, NTSR1 can also form extrasynaptic heterodimers with the D_2_ dopamine receptors to blunt their ability to inhibit dopamine release while also possibly promoting PKC activity (see [92]).

There are four ligands that arise from the neurotensin precursor (*NTS*), large neuromedin N (l-NN), large neurotensin (l-NT), NN and NT, although within the brain NT and NN are the predominant ligand variations [143]. Like many of the neuropeptide transmitters neurotensin requires cleavage from a prohormone, so while the local nigral volume contains low levels of *NTS*, it contains proportionately more transcript of the proprotein convertases; PC1 (*PCSK1*), PC2 (*PCSK2*) and PC5A (*PCSK6*) which regulate the relative proportions of NN and NT according to their expression. Thus PCSK2 protein produces equal amounts of NN and NT, while PCSK1 and PCSK6 favour NT [143]. Within the pigmented neurons, *PCSK2* and *PCSK1* transcripts are the most abundant and likely selectively expressed in pigmented neurons since there is a significant loss of both transcripts within the nigral volume. In contrast, *PCSK6* is barely expressed in pigmented neurons and is significantly elevated within the nigral volume. With little *NTS* transcript in the pigmented neurons it seems logical to suggest that the PCSK1/2 proteins are used in the processing of other prohormones (see [144]). Whether the *PCSK* gene changes in the nigral volume result in different *NTS* products remains to be seen. As a point of comparison, the caudate nucleus contains the highest levels of *NTS*, *PCSK2* and *PCSK6* transcripts across the four regions of measurement (**Fig 4**). Once released *NTS* products are broken down by a number of endopeptidases including membrane metalloendopeptidase (*MME*), thimet oligopeptidase (*THOP1*) and neurolysin (*NLN*) [145]. Of these *THOP1* and *NLN* are the most abundantly expressed in both pigmented neurons and nigral volume and this expression does not change with PD (**Fig 4**).

In addition to its own agonist responses, NTSR1 can associate with NTSR3, also called sortilin (*SORT1*); a type I Vps10p-containing domain receptor family member that is also a receptor for pro-forms of nerve growth factor (*NGF*) and neurotrophins (*NTF3/4*) [146]. However, the pigmented neurons, nigral volume, caudate nucleus or putamen contain little transcript for these ligands (**S2 Table**).

***HTR2A*** codes for the serotonin (5HT) 2A receptor. While relatively abundant in pigmented neurons, the key enzymes in the synthesis of 5HT; tryptophan hydroxylases 1 and 2 (*TPH1* and *TPH2*, respectively) are very weakly expressed both within the pigmented neurons and within the substantia nigral volume. This is not unexpected since the primary source of nigral 5HT likely originates from the dorsal and medial raphe nuclei [147].

The angiotensin II type 1 receptor (***AGTR1***) is modestly expressed in neurons. The receptor promiscuously couples Gα_i/o/q/11_ G-protein subunits and is particularly interesting since the nigral volume contains a substantial level of precursor, angiotensinogen transcript (*AGT*). There are also modest levels of AGT expressed within the pigmented neurons which is consistent with reports of the presence of the angiotensin receptor and angiotensinogen in human nigral neurons [148]. However, there appear to be some unknowns with respect to the origin and role of angiotensin signalling within the substantia nigra. Thus, within the classically described renin-angiotensin system, angiotensinogen is processed to angiotensin I, predominantly by renin (*REN*), and then to angiotensin II by angiotensin converting enzymes 1 and 2 (*ACE1* and *ACE2*, respectively). There is, however, hardly any transcript for these enzymes in either the nigral volume or pigmented neurons (**Fig 4**). As an alternative, a scheme has been proposed where plasma borne pro-renin can be processed to renin by the peptidases cathepsin B and D (*CTSB* and *CTSD*, respectively); renin (*REN*) or even cathepsin D (*CTSD*) can then process angiotensinogen to angiotensin I. However, this scheme relies on prorenin crossing the blood brain barrier since there appears to be little transcript within pigmented neurons or the nigral volume. There is, however, an abundance of *CTSB* and *CTSD* within the nigral volume, and also within pigmented neurons (**Fig 4**) indicating the potential for a mechanism to generate local angiotensin I [149]. Besides ACE, there are multiple enzymes that can convert angiotensin I to angiotensin II these include chymase (*CMA1*) [150], elastase-2 (*ELANE*) [151], tissue kallikreins (likely *KLK1*) [152] and cathepsin G (*CTSG*) [149]. These enzyme transcripts are however, with the exception of *KLK6*, only weakly expressed in the nigral volume or neurons (**Fig 4**, **S2 Table**) indicating that perhaps angiotensin II is not the primary signalling product of angiotensin I. An alternative product, angiotensin [1–7] can be produced from angiotensin I by neprilysin (*MME*), thimet oligopeptidase (*THOP1*) and prolyl endopeptidase (*PREP)* [153,154]. Of these *THOP1* and *PREP* are expressed at low levels within the nigral volume, although the PREP related, Prolyl Endopeptidase Like protein (*PREPL*) is far more robustly expressed, whether it plays a role in angiotensin function is currently unclear. Regardless of potential production mechanisms, a role for angiotensin [1–7] in pigmented neurons seems unlikely since the cognate receptor for angiotensin [1–7], MAS1 Proto-Oncogene, G Protein-Coupled Receptor (*MAS1*) [155] is barely detectable within the nigral volume or pigmented neurons. Another product of angiotensin II, angiotensin III (angiotensin (2-8)) is produced through the action of aminopeptidase A (*ENPEP*) [156], once again, however, transcript for this enzyme is scarcely present (**S2 Table**). While angiotensinogen transcript is modestly expressed in pigmented neurons, it is robustly expressed within the nigral volume and highly expressed within the caudate nucleus and putamen. Identifying the precise metabolic fate of local angiotensinogen remains a challenge, given that its known processing enzymes are so poorly represented within the nigral transcriptome.

#### 3.6.1. Transduction processes.

At the simplest level, the activation of Gα_q/11_ (*GNAQ* and *GNA11*) family members will stimulate phospholipase Cβ isoforms (*PLCB1/2/3/4*). For the pigmented neurons PLCβ1 and PLCβ4 are the most abundant isoforms (**S2 Table**). These enzymes cleave phosphoinositol bisphosphate (PIP_2_) to generate diacylglycerol (DAG) and inositol trisphosphate (IP_3_) both of which have the capacity to interact with intracellular signalling components. Once released from PIP_2_, IP_3_ activates ligand gated ion channels present on intracellular calcium stores (*ITPR1/2/3*), where the predominant pigmented neuron IP_3_ receptor transcript is *ITPR1* (**S2 Table**). Released calcium will have a major impact as a signal within the cells either directly, for example binding to conventional isoforms of protein kinase C (*PRKCA/B/G*), or indirectly through binding to calmodulin (*CALM1/2/3*) and subsequent activation of downstream effectors such as calmodulin kinases (*CAMK2*s). The calmodulin kinase transcripts are abundantly expressed in the mammalian CNS but also subject to alternative splicing (see [157]). These transcripts are highly expressed in pigmented neurons, but somewhat surprisingly the most abundant isoform in the mammalian CNS, *CAMK2A*, is the most poorly expressed in the pigmented neurons, which have an abundance of transcript for Ca^2+^-calmodulin dependent kinase II isoforms 2B, 2D & 2G (*CAMK2B/G/D*). That the most abundant CAMK2 isoform in pigmented neurons, *CAMK2B,* is significantly downregulated in the nigral volume may indicate that this isoform is localized to nigral neurons (**S2 Table**). Functionally, calmodulin kinase II proteins contribute to the regulation of diverse cellular processes including dendritic spine plasticity via monomeric G-proteins (*RHOA* and *CDC42*) [158], the regulation of P/Q voltage-gated calcium (*CACNA1A*), AMPA (*GRIA1* subunit) [159] and GABA_A_ receptor subunit activity [160] as well as the positive or negative regulation of IP_3_ receptors [161]. Perhaps most significantly, CAMK2 has been shown to inhibit, via phosphorylation, GSK3α and β activity (*GSK3A* and *GSK3B*, respectively) to promote cell survival [63] (**Fig 4**). Thus, any disruption to Gα _q/11_-CaMK2 signalling is likely to promote the pro-apoptotic actions of uninhibited GSK3β during Parkinson’s disease progression.

The calcium-calmodulin complex will also activate other calmodulin-dependent kinases, including Eukaryotic Elongation Factor 2 Kinase (*EEF2K*), expressed calmodulin-dependent kinase kinases 1 and 2 *(CAMKK1/2*) and the kinase-like protein, CaM Kinase Like Vesicle Associated (*CAMKV*). While the transcripts are not expressed at high levels, CAMKK1/2 play important roles in the regulation of other kinases, for example, Calcium/Calmodulin Dependent Protein Kinase I (*CAMK1/1D/1G*) isoforms, Calcium/Calmodulin Dependent Protein Kinase IV (*CAMK4*), AMP-kinases (*PRKAA1/2*) and AKT Serine/Threonine Kinases (*AKT1/2/3*) (see [162]). In addition, they are subject to inhibition by PKA (*PRKA* isoforms) which phosphorylates CAMKK1 to enable 14-3-3 protein (*YWHAZ*) recruitment and stabilization of the inactive form of the kinase [163]. While not a kinase, calmodulin kinase-like vesicle-associated (*CAMKV*) is localized to dendritic spines where it binds to and inhibits Rho/Rac Guanine Nucleotide Exchange Factor 2 (*ARHGEF2*) to prevent RHOA-dependent spine retraction; this protective mechanism is suppressed when cyclin dependent kinase 5 (*CDK5*) phosphorylates CAMKV, preventing its interaction with ARHGEF2 [164], (**Fig 4**).

Elevated intracellular calcium and diacylglycerol, or diacylglycerol alone, will also activate the modestly expressed conventional (*PRKCB, PRKCG, PRKCA*) and novel PKC (*PRKCD*) isoforms, respectively (**Fig 4**). In turn, PKC can then potentiate N- (*CACNA1B*) and P/Q-type (*CACNA1A*) channel activity while also curtailing the ability of Gβγ subunits to bind to and inhibit these channels [165]. Similarly, PKC can inhibit phospholipase Cβ (*PLC1−4*) activity [166], either by phosphorylating PLC itself, or the Gβγ subunits to prevent their binding. In addition to regulating calcium channel activity, PKC (conventional and atypical) can phosphorylate, and thereby inhibit the activity of the Raf kinase inhibitory protein (*RKIP*), promoting Raf pathway activity [167]. Interestingly, the most abundant of the Src family kinases present in the pigmented neurons, FYN Proto-Oncogene, Src Family Tyrosine Kinase (*FYN*) is capable of both activating Raf-1 Proto-Oncogene, Serine/Threonine Kinase (*RAF1*) [168], as well as modifying PKCδ (*PRKCD*) [169]. While Src-mediated tyrosine phosphorylation of PKCδ is traditionally associated with kinase inactivation in keratinocytes [169], in neurodegenerative contexts, FYN-mediated phosphorylation of PKCδ (Y311) triggers pro-apoptotic activity [170], **Fig 4**.

PIP_2_ can also interact directly with voltage gated ion channels to regulate their activity, including a bidirectional control of Cav2.1 (*CACNA1A*), inhibition of the shaker related potassium channel family member Kv1.2 (*KCNA2*) [171], enhancement of the stimulatory action of Gβγ subunits on GIRKs (such as *KCNJ6*) [172] and opening Kir2.2 (*KCNJ12*) channels [173]. The current analysis indicates little basis for a change in PIP_2_ levels in pigmented neurons since their synthetic machinery transcripts (*CDIPT*, *CDS2* and *CDS1*) do not change in PD (**Fig 4**).

Clearly, modulation of Gα_q/11_ family member c oupled GPCRs seems likely to have an impact upon cell morphology and survival mediated particularly through the modulation of monomeric G-protein family and calcium-dependent kinase signalling. There is, however, little evidence of change within Gα_q/11_ dependent GPCR signal transduction within the pigmented neurons. Thus, if Gα_q/11_ signalling changes in PD, it seems likely mediated via signal inputs to the nigra. Identifying the source of, and potential changes in ligands stimulating the neurotensin-1, 5HT_2_ family members and angiotensin receptors may make future assessments of Gα_q/11_ signalling outcomes in pigmented neurons more predictable.

### WNT receptor signalling

**Fig 5:**
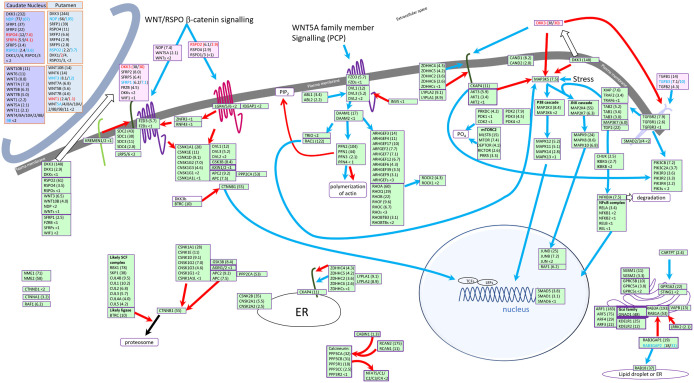
Speculative WNT family signalling pathways in pigmented neurons. The image includes gene symbols for transcripts for ligands presumed present in the nigral volume and/or synaptic spaces (pink boxes), putamen or caudate nucleus (mauve and blue boxes, respectively) and the pigmented neurons (green boxes). The numbers in the brackets represent the transcript per million (TPM) from control tissue/cells. If the differential expression analysis (DEA) of the data set shows a significant change transcript associated with Parkinson’s disease, then the gene name is coloured red for a decrease or blue for an increase. The significant change in expression (control TPM adjusted by DEA value) is represented by the number after the forward slash (/). The white boxes (for example, the one containing PIP_2_) generally indicate a process or substance produced, introduced into or activated within the cellular space. The red arrows indicate an inhibitory action while the blue arrows indicate a stimulatory action (sometimes blue and red arrows connect the same pathways to indicate context dependent signalling). The white arrows indicate a potential for substance movement across membranes. When boxes are connected it indicates a likely binding partner (AXIN and APC, for example). Some transcripts, for example some of the G-protein coupled receptor kinases (GRKs), are weakly expressed and listed as other GRKs < 2, indicating that remaining family member transcripts are expressed at less than 2 TPM (**S2 Table** shows all transcript values). This is a stylized map of activity it is not intended to define subcellular or the extracellular locations of transcript derived proteins with any degree of accuracy. See text for details of major pathway interactions and definitions.

Historically, Wnt signalling was divided into β-catenin-dependent and β-catenin-independent signalling pathways which are activated by 10 largely Wnt sensitive class F (Frizzled) seven transmembrane spanning receptors (*FZD1–10*). The frizzled receptors offer distinct sets of activities not necessarily included with the other GPCRs. Of the Frizzled receptors, Frizzled-3 (FZD3) is the most abundant transcript within the pigmented neurons and has been linked to pre-synaptic regulatory actions [174]. It, along with FZD6, is efficiently stimulated by subset of WNTs, in particular WNT5A type ligands (*WNT4, WNT5A, WNT5B, WNT6, WNT7A* and *WNT11*), and appears to be particularly effective in activating the β-catenin-independent planar polarity pathway (PCP, see [175]). FZD receptor activation of the PCP (and Ca^2+^-dependent signalling), usually involves any of a number of co-receptors, including protein tyrosine kinase 7 (*PTK7*), muscle-skeletal receptor tyrosine kinase (*MUSK*), tyrosine kinase-like orphan receptors (*ROR1* & *ROR2*), tyrosine kinase related receptor (*RYK*), the heparan sulfate proteoglycan syndecans (*SDC1–4*) and glypicans (*GPC1–6*), as well as the planar cell polarity proteins (*PRICKLE1–4*) and/or VANGL Planar Cell Polarity Proteins 1 and 2 (*VANGL1/2*) (see [176]). Frizzled receptor activation can promote alternative cellular activities, particularly in *Xenopus*, where Fzd3 triggers the recruitment of Dishevelled (Dvl1–3) [175,176]. This recruitment leads to distinct downstream cascades; one branch involves Dvl association with Dvl-associated activator of morphogenesis (*DAAM1/2*), which activates RhoA via Rho/Rac Guanine Nucleotide Exchange Factors 2 and 18 (*ARHGEF2/18*) [178], subsequently targeting Profilin (*PFN1–4*) to modulate actin monomer dynamics [177]. Concurrently, a parallel branch drives Rac1 (*RAC1*) activation, which can be mediated by the Trio Rho Guanine Nucleotide Exchange Factor (*TRIO*) [176], **Fig 5**.

Activation of the β-catenin-dependent pathway begins with Wnt ligands binding to Frizzled (FZD) receptors and low-density lipoprotein receptor-related protein (*LRP5/6*) co-receptors. This enables dishevelled (*DVL1/2/3*) binding to, and inhibition of, the β-catenin destruction complex: glycogen synthase kinase 3β (*GSK3B*), adenomatosis polyposis coli (*APC*), axin1 (*AXIN1*) and casein kinase 1α (*CSNK1A1).* It is interesting to note that the pigmented neurons express little *AXIN1* or *AXIN2*. Overexpression of Axin promotes a degradation of β-catenin levels [177] highlighting the possibility that β-catenin levels in pigmented neurons might be tonically higher than expected due to low axin expression. Just as a point of comparison, while *AXIN1* transcript is very poorly expressed in pigmented neurons, it is more robustly found, and significantly increased, in the PD nigral volume. In comparison, *AXIN1* shows much greater expression in both caudate nucleus and putamen (**S2 Table**). In the absence of Wnt signal, β-catenin is destroyed by the proteasome via an interaction with the F-box containing E3-ligase β-TrCP (*BTRC*), S-Phase Kinase Associated Protein 1 (*SKP1*) and the ligase cullin-1 (*CUL1*) to attach ubiquitin to its binding partners (see [178]), **Fig 5**.

Despite low levels of FZD and LGR receptor transcripts, WNT signalling seems likely to be of importance to pigmented neurons since they generate so much transcript related to its regulation; namely roof plate-specific spondin-2 (*RSPO2*) and dickkopf-3 (*DKK3*), **Fig 5**. RSPO2 is one of several regulators of the WNT signalling pathway [179], it is highly expressed within pigmented neurons but also significantly reduced in the nigral volume. It is generally considered as a WNT β-catenin pathway agonist, where it binds to leucine-rich repeat-containing receptors, Lgr4/5/6 (*LGR4/5/6*) to prevent the ligase, Zinc And Ring Finger 3 (*ZNRF3*) ubiquitylating FZDs, thereby preventing FZD degradation. Alternatively, RSPO2 can also bind to the scaffold protein IQ Motif Containing GTPase Activating Protein 1 (*IQGAP1*) to drive Wnt–β-catenin signalling directly (see [180]). It is interesting to note that *ZNRF3* expression is very low in pigmented neurons indicating a resistance to FZD degradation (promoting both canonical and non-canonical signalling). Rspo2 has been suggested, in *Xenopus* studies, to inhibit both WNT signalling, by inhibiting Transcription Factor 3 phosphorylation (*TCF3*) [181], as well as fibroblast growth factor induced Erk1 activity [182]. Generally, within the pigmented neurons, mechanisms that might switch off β-catenin signalling are poorly represented; noting the very low expression of *AXIN*, *ZNRF3* and inversin (*INVS*), which targets dishevelled (*DVL1/2/3*) for ubiquitinylation [183]. These data indicate that pigmented neurons have a capacity to tonically regulate β -catenin and perhaps other signal transduction mechanisms via the high expression of *RSPO2* transcript. If that is the case, then it will be interesting to see how a substantial reduction (> 50%) of this transcript in the nigral volume impacts neuron survival, and the identity of the signal transduction mechanisms employed.

Dickkopfs are a family of four secreted glycoproteins (*DKK1−4*). Dickkopfs 1/2/4 bind to frizzled co-receptors; Lrp5/6 (*LRP5/6*) and Kremen 1/2 (*KREMEN1/2*) to facilitate endocytosis to antagonize WNT signalling. In contrast, Dickkopf 3 activity is not as clearly defined and there is evidence indicating a more nuanced role for it to both positively and negatively impact WNT signalling (see [184]). A recently delineated and, in the context of this work, particularly interesting pathway involves Cytoskeleton Associated Protein 4 (*CKAP4*), a protein originally identified as type II transmembrane protein anchoring the endoplasmic reticulum to microtubules [185]. More recently, a portion of the total CKAP4 protein has been identified in the plasma membrane; subject to regulation following S-acylation by ZDHHC palmitoyl transferases (*ZDHHC2/6/5*) and de-acylation by Lysophospholipase 2 (*LYPLA2*) [186]. The membrane bound CKAP4 is a binding partner for both DKK1 [187] and DKK3 ([188]) which permits Akt (*AKT1/2/3*) phosphorylation [189] to activate NFκB and cell proliferative pathways (see [184]) while inhibiting upstream JNK (*MAP2K4*) signalling [190] (**Fig 5**). While *CKAP4* is modestly expressed in neurons (and the nigral volume, **S2 Table**), DKK3 might also possess alternative activities. It can, for example, inhibit TGFβ receptor-1/2 expression (*TGFBR1/2*) and or signalling [191,192]. While TGFβ receptors can signal through activation of the canonical Sma- And Mad-Related Proteins 2,3 and 4 (*SMAD2/3/4*) pathway, or via a ubiquitin ligase (*TRAF2/6* or *XIAP*)/ TGF-Beta Activated Kinase 1 (*MAP3K7*) Binding Protein (*TAB1/2/3)* complex [193] to stimulate Mitogen-Activated Protein Kinase Kinase Kinase 7 (*MAP3K7*) activity leading to the activation of JNK, p38 Map kinase pathways or the stimulation of NFκB (*NFKB1/2*) signalling. For the latter, briefly, TAK1 in complex with TAB proteins, phosphorylates the IKK complex (inhibitor of nuclear factor Kappa B kinase (*CHUK*)- inhibitor of Nuclear Factor Kappa B Kinase Subunit Beta-Gamma (*IKKB/IKKG*)) to subsequently phosphorylate IκBα (*NFKBIA*) to alleviate repression of the NFkB complex which can then translocate to the nucleus to support cell survival [194], **Fig 5**. In addition, TGFβ signalling may also activate Ras Homolog Family Member A (*RHOA*) and PI3 kinase (*PI3K* components) [195].

DKK3 also impacts signalling through an N-terminally truncated, cytoplasmic form, DKK3b, that binds to the Beta-Transducin Repeat Containing E3 Ubiquitin Protein Ligase (BTRC) and unphosphorylated β-catenin to prevent nuclear translocation, while also inhibiting the proteasome-mediated degradation of β-catenin [196]. Additionally, DKK3 may have the capacity to bind directly to and inhibit the activity of MAP3K5 (Apoptosis Signal Regulating Kinase 1) a key mediator of cardiac hypertrophy [197] and the starting point for the activation of stress-related apoptosis (see [198]), **Fig 5**.

Generally, DKK3 protein is protective of neurons (see [199]) thus it makes sense that, regardless of the mechanism, a reduction in its expression may promote stress in neurons. In what seems likely to complicate nigral volume reductions in *DKK3* and *RSPO2* is an elevation of the WNT-reducing extracellular signal, secreted frizzled related protein-1 (*SFRP1*) transcript. While originally defined as WNT-signalling antagonists, SFRP proteins have more wide-ranging functions, from the activation of FZD receptors to promote heterotrimeric G-protein activity, to the modulation of integrin-fibronectin complex, to binding to the tumor necrosis factor family member, Receptor Activator Of Nuclear Factor Kappa B Ligand (*TNFSF11*), see [200]. *SFRP1*, along with other members of SFRP family (*SFRP2, 4* and *5,* but not *FRZB* (formerly *SFRP3*)) are all clearly expressed within the nigral volume, at higher levels than present in pigmented neurons; perhaps indicating that these signals are not secreted predominantly from dopaminergic neurons.

Note that some of the Frizzled receptor signalling pathway effects can be potentiated by R-spondin (*RSPO1–4*)-sensitive leucine-rich repeat-containing receptors (*LGR4/5/6*) possibly through interaction with membrane-bound heparan sulfate proteoglycans (HSPGs) such as syndecans (*SDC1–4*) [179]. The nigral volume also contains comparatively high levels of Norrin Cystine Knot Growth Factor (*NDP*), a ligand know to activate frizzled-4 receptors [201], although that seems unlikely in this context given the very low levels of *FZD4* present in the pigmented neurons.

Overall, these changes in WNT-related signalling, in particular the nigral volume reduction in *DKK3* seem likely to have an adverse impact upon pigmented neurons since WNT-related signalling plays such crucial roles in both dopaminergic neuron development [202–205] and survival (see [199]). With respect to likely pathways, the low transcript levels for components of the *SMAD2/3/4* and *CHUK/IKKB/G* complex indicate that p38 and JNK pathways, perhaps along with PI3 kinase and RhoA pathways appear most likely to be impacted by any potential loss of DKK3 from the nigral volume. Elucidating the roles, if any, of CKAP4, MAP3K5, XIAP and DAAM1 in promoting neuron survival may illustrate a potential mechanism for the contribution of DKK3 to pigmented neuron health and survival.

### Other GPCR and related signalling activities

While the focus of this work is on traditional cytoplasmic membrane bound GPCR signalling, members of this receptor family have been found on intracellular organelle membranes where they can still signal through heterotrimeric G-proteins. For example, transcript for G Protein-Coupled Receptor Class C Group 5 Member B (*GPRC5B*) and two of the KDEL Endoplasmic Reticulum Protein Retention Receptor subfamily members (*KDLER1* and *KDELR2*) are expressed in pigmented neurons. GPRC5B protein is strongly localized to the Golgi network where it interacts with sphingomyelin synthase 2 (*SGMS2*) [206] and has been linked to mood and anxiety disorders [207]. The seven transmembrane spanning KDELR proteins have a role in regulating intracellular trafficking where they respond to molecular chaperones containing a KDEL sequence. These receptors interact with Golgi Gα_o_ proteins to activate (at least) Rab3a (*RAB3A*) via a Golgi bound GEF to potentiate plasma membrane-directed trafficking linked to the elongation and stability of membrane protrusions [208]. It’s interesting to note that *RAB3GAP2*, which encodes the regulatory subunit of the RAB3GAP complex, but not *RAB3GAP1* (the catalytic subunit) transcript is significantly elevated in pigmented neurons (**Fig 5**). The complex serves to increase the GTPase activity of RAB3 monomeric G-protein subfamily members, including the protein for the very abundantly expressed *RAB3A* transcript [209], and appears sensitive to inhibition by Leucine Rich Repeat Kinase 2 (*LRRK2*), which is weakly expressed [210]. In contrast, the RAB3GAP complex acts as a GEF for the protein of the robustly expressed *RAB18* transcript, where it helps maintain endoplasmic reticulum tubular networks [211], but is also found in lipid droplets [212]. Thus, Rab3 and Rab18 are regulators of endomembrane trafficking dependent upon the RAB3GAP complex. Additionally, the RAB3GAP1/2 complex also regulates autophagy [213], the degradation of cellular components in lysosomes (**Fig 5**). Critically, a failure of autophagy results in neuron death [214] and mutations in RAB18, RAB3GAP1 and/or RAB3GAP2 are associated with Warburg Micro and Martsolf syndromes [215]. At present it is not clear what impact, if any, that an increase in one component of the RAB3GAP1/2 complex would have on function and survival in dopaminergic neurons, but it would be very interesting to find out.

#### 3.8.1. Gβγ-driven transduction.

While the Gα subunits regulate much cellular activity, the Gβγ subunits, liberated from GTP-bound Gα subunits following agonist binding to GPCRs, also play substantial roles in regulating cellular activity. Within the pigmented neurons β_1_γ_3_ and β_2_γ_3_ seem likely to be the most abundant G-protein βγ subunits dimers, although β_5_ and both γ_10_ and γ_2_ are robustly expressed. While not necessarily being linked with specific combinations, βγ subunits can inhibit calcium channels [216], activate PLA_2_ [217] and stimulate, in the presence of PIP_2_, G-protein gated inward rectifying K^+^ (*KCNJ* family) channels [218]. Gβγ can also directly activate PI3Kβ(*PIK3CB*) to drive proliferation and survival [219]. Gβγ subunits can interact with a histidine kinase complex (*NME1-NME2*), where Gβ subunits are both a binding partner and substrate for the kinase, which may then phosphorylate histidine residues on a number of proteins, including Intermediate Conductance Calcium-Activated Potassium Channel Protein (*KCNN4*) (see [220]). Lastly, the βγ subunit of heterotrimeric G proteins can be either stimulatory or inhibitory depending on the specific adenylate cyclase isoform. Of these, the effects of Gβγ appears to be inhibitory for ADCY3 and stimulatory for ADCY2 and ADCY5 (see [221,222]). Since *ADCY2/3/5* represent over 80% of the pigmented neuron ADCY transcripts it seems possible that, given the loss of *DDC* and *TH* from the nigral volume, that there is a reduced capacity to activate PKA and or PI3Kβ in pigmented neurons via reduced dopamine liberation of Gβγ. For more potential interactions see [223].

### Are there common elements regulating the changes in gene expression?

There are many changes in transcript expression within the nigral volume, a finding perhaps not surprising given the likely change in nigral cell profile due to the loss of pigmented neurons. There are, however, far fewer changes in pigmented neuron transcript expression: two downregulated and 30 up-regulated genes. Filtering those up-regulated transcripts to include those with something more than negligible expression (an arbitrary >2 TPM) produces a list of nine upregulated transcripts: *HSP90AA1*, *HSPA1B, RAB3GAP2*, *XBP1*, *ZMYND8*, *HSPA1A*, *SLC5A3*, *ZNF331* and *L3MBTL1*. A ChEA3 analysis of these nine up-regulated transcripts produces a list of 11 transcription factors that can, individually, regulate expression of up to seven of the nine upregulated transcripts: *JUND, STAT3, YY1, TBP, NFYB, CREM, NFIC, ELK1, E2F1, ATF3* and *MYBL2* (**S4 Table**).

Within the pigmented neurons JUND and STAT3 are by far the most abundant transcripts present (25 and 12 TPM, respectively). JUND can be activated via c-Jun N-terminal kinase [224,225] and PKC-ERK pathways [226] while STAT3 signal transduction occurs via the Janus kinase (JAK) family. The next three most abundant TFs: *YY1* (7 TPM), *TBP* (6 TPM) and *NFYB* (6 TPM) are likely regulated through Src family kinases [227], Ras/Raf activity [228] and non-canonical NFκB signalling [229], respectively. While the focus of this work on GPCRs is limiting with respect to the pathways activated there is enough evidence, based on transcriptomic studies, to suggest some potential interaction between the GPCR signal transduction pathways identified and the transcription factors regulating expression of the overexpressed genes. For example, Gβγ subunits can activate MAP2K4, and therefore JNK signalling, via a likely interaction with CDC42 and or RHO, in a phosphatase dependent manner [103–105,108]. Alternatively, DKK3 inhibits MAP3K5 via multiple mechanisms [190,193,197] thereby mitigating the activation of upstream JNK signalling and stress-related apoptosis. (see [198]). At the level of transcript changes these data might predict a hypothesis where loss of DKK3 (via reduced *DKK3* expression) leads to disinhibition of stress-related (JNK-mediated) pathways, placing pigmented neurons under additional stress. Whether this effect might outweigh the impact of potentially reduced Gβγ stimulation is unknown but given that PD is a disease that spans decades, subtle alterations in the balance of pro- vs anti-survival signalling, over an extended period of time, might be enough to initiate a slow decline of pigmented neuron numbers.

Lastly, while lacking transcript for its GPCR target receptor (*CX3CR1*), the pigmented neurons contain moderately high levels of fractalkine (*CX3CL1*) which significantly declines within the nigral volume with PD (**S2 Table**). Early studies showed that fractalkine was a membrane bound signalling protein (see [230]); it can, however, be released from cells through action of ADAM10 (*ADAM10*), ADAM17 (*ADAM17*), MMP2 (*MMP2*), and cathepsin S (*CTSS*) (see [231]). Note that these enzymes are all weakly expressed by pigmented neurons, although *ADAM10* is most abundant in the nigral volume (**S2 Table**). These data indicate that CX3CL1 may bind directly to CX3CR1 or be processed locally within the nigral volume to reduce microglial activation. Activated microglia (M1 phenotype) are defined by upregulation of specific markers, including; *TSPO, ITGAM, MMP9*, *CD14* and *FCGR3A* [232,233]. Of these markers, *ITGAM* (5 TPM), MMP9 (<1 TPM) and TSPO (18 TPM) are all significantly upregulated perhaps indicating a low level, or substate of nigral microglial activation, perhaps linked to the initiating stimulus [234]. *CX3CL1* is also reduced in PD putamen and caudate nucleus (**S2 Table**). Curiously, neuroprotective ligand transcripts such as *IGF1* and *BDNF* [234] are weakly expressed in the nigral volume, with *BDNF* significantly lower in PD (**S2 Table**).

## Discussion

The current study maps PD-related changes in local and distal GPCR signal-related transcripts and matches these to changes in pigmented neuron GPCR and signal related transduction mechanism transcripts, primarily with the purpose of identifying the PD-dependent conditions that the pigmented neurons are likely subject to within the substantia nigra pars compacta. This study has used transcriptomic data, both differential expression analysis and RNAseq to identify changes, and the magnitude of those changes, in nigral volume and pigmented neurons along with changes in caudate nucleus and putamen. Overall, there are surprisingly few changes in pigmented neuron gene expression; although it should be noted that these significantly dysregulated genes are identified with an arbitrary Q value of 30%, indicating the possibility that approximately 30% of the significant values are likely false positives. Increasing Q to 40% increases the number of “significantly” altered genes to include *RHOQ*, *CREB3* and *CALB1* while decreasing Q to 3%, as it is for the larger “n” nigral volume analysis, means that no genes are significantly dysregulated by PD in pigmented neurons. That there are fewer changes in gene expression evident across the pigmented neurons than there are across the nigral volume indicates that most of the nigral volume transcript changes can be attributed to the change in cellular composition of the nigral volume, i.e., as the pigmented neurons die-off they contribute less to the pool of transcripts.

While the data indicate multiple changes both within pigmented neurons and more broadly within the nigral volume there are three sets of changes that seem likely to have major impacts upon neuron function. These are changes in the robust signalling mechanism transcripts representing Gα_o_-directed (which includes βγ- activity), DKK3-mediated signalling and perhaps also fractalkine (CX3CL1) signalling.

Gα_o_ is the third most abundant of the G-protein alpha subunits (behind Gα_s_ and Gα_q_ family members) present in pigmented neurons and has been linked to a variety of signalling pathways in different cell systems. For example, it has been shown to regulate the activity of the GTPase activating protein, RAP1GAP [235] and the monomeric G-protein, Rho [236]. It has also been linked to the activation of MAPK and NFkB in mast cells [237] as well as the regulation, via liberated βγ subunits, of adenylate cyclase activity [238]. Because *GNAO1* is abundantly expressed in the brain, Feng and co-workers investigated its clinical relevance, reporting that loss-of-function mutations are associated with epileptic encephalopathy, whereas gain-of-function mutations are tied to movement disorders [98]. Although that study relied on a basic cAMP accumulation assay to classify these mutations, it highlights the critical role of stable *GNAO1* signalling in modulating neuronal function. Further work by Benedetti and colleagues, using neurons derived from induced pluripotent stem cells shows that the loss-of function G203R *GNAO1* mutation [239] reduces the spontaneous activity of IPSC derived neurons, sensitivity to neurotransmitters and, late in differentiation, the presence of (Ser9) phospho-GSK3β [240]. Crucially, dopamine D_2_ receptor directed Ser9 phosphorylation of GSK3β, via β-arrestin and AKT, reduces kinase activity, promoting β-catenin translocation and downstream signalling [241]. Additionally, AKT suppresses the activation of stress-related JNK signalling [190]. Collectively, these insights suggest that reduced nigral dopamine leads to both reduced Gα_o_ signalling and/or dopamine-driven D_2_ receptor internalization and β-arrestin activation (likely impacting β-catenin, ERK and AKT signalling) within pigmented neurons. Consistent with the idea of reduced availability of dopamine for pigmented neuron feedback, Gröger & co-workers have shown reduced nigral dopamine content associated with Parkinson’s disease [242]. While some of these D_2_ dopamine receptors are certain to be pre-synaptic, Gα_i_-coupled, some will also serve as post-synaptic mediators of dopamine feedback activating the more abundant members of the Gα_i/o_ G-protein family [91], in particular Gα_o1_. There are, however, multiple transcripts for receptors capable of Gα_o_ signalling in pigmented neurons. Thus, compounding the likely impact of lowered nigral dopamine signalling, these neurons also show significantly reduced expression of *SSTR1*.

Given the capacity of dopamine receptors to modulate β-catenin signal transduction, it is noteworthy that two canonical Wnt signalling ligands—*DKK3* and *RSPO2*—are among the most highly expressed signal-related transcripts within pigmented neurons. Crucially, the nigral expression of these specific transcripts is markedly reduced in Parkinson’s disease (PD). Wnt signalling plays an essential role in dopaminergic neuron development by regulating both early- and late-stage transcription factors, including *EN1/2*, *OTX2*, *FOXA2*, *LMX1A*, *MSX1*, *NEUROG2*, *ASCL1*, *NR4A2*, and *PITX3* [243–247]. Of these *EN1*, *NR4A2*, *FOXA2* and *PITX3* transcripts are all highly expressed with pigmented neurons (**S2 Table**). *PITX3* expression is critical for defining mature midbrain dopaminergic neurons due to its highly restricted localisation in the midbrain [248], particularly within the ventral substantia nigra [249]. In this region, PITX3 facilitates tyrosine hydroxylase (TH) expression, likely by de-repressing *NR4A2* activity [250]. Thus, while Wnt ligands dictate the adoption of a dopaminergic phenotype during development, they also promote mature cell survival [251–253]. Consistent with our current findings, *RSPO2* is reportedly downregulated in PD substantia nigra tissue [254], suggesting that both *RSPO2* and the more abundantly expressed *DKK3* are predominantly produced by dopaminergic neurons. We previously demonstrated that β-catenin regulates TH expression in pluripotent stem cell-derived, dopaminergic-enriched cultures and suggested this effect was mediated by the transcriptional repressor *NR0B1*, which suppresses the expression of the Wnt/β -catenin activator RSPO2 [255]. In the current dataset, however, *NR0B1* transcripts were undetectable across most RNA-seq samples. This might reflect low baseline expression levels consistent with its function as a transcriptional repressor. Regardless, these data underscore a capacity for widespread dysregulation across multiple signalling networks, particularly those governed by Gα_o_ and DKK3*/*RSPO2. Compounding the likely loss of these signals from the nigral volume is a reduction in fractalkine transcript (*CX3CL1*) which may, in turn, contribute to microglial activation.

While considerable research activity and many therapeutic interventions focus on maximizing striatal dopamine activity, comparatively little is known about the local, non-neuronal feedback networks that support neuronal activity within the substantia nigra. This study utilises existing transcriptomic profiling studies to identify and quantify changes in these local signalling systems, at least with respect to likely activators of G-protein coupled receptors, assessing the possible impact of changes based on relative levels of expression. Beyond the focus on Gα_o_ and WNT signalling regulators many questions must be addressed before we can begin to suggest an understanding of local nigral signalling activity. For instance, how closely do transcript levels mirror protein expression? There are several orphan receptor transcripts clearly expressed: GPR158, GPR162, GPRC5B, GPR137, however, their purpose and ligands remain unclear. Does the relatively weak expression of fractalkine in the nigral volume make this region more susceptible to pigmented neuron death and microglial activation than striatal tissue? *ZMYND8* expression can be increased by hypoxia [256], oxidative stress/ferroptosis [257] and the unfolded protein response [258]. It is also increased in PD-surviving pigmented neurons, can this effect be linked to JUND, STAT3 or even GSK3β? Finally, is angiotensinogen locally converted to angiotensin II, or one of the smaller signalling fragments?

*RAB3GAP2* transcript codes for the regulatory subunit of the Rab3 GTPase-activating (Rab3GAP) complex (*RAB3GAP1* and *RAB3GAP2*), which has GTPase-activating protein (GAP) activity towards various Rab3 subfamily members where it modulates neurotransmission and plasticity [259]. Does increased *RAB3GAP2* transcript lead to altered RAB3GAP1 protein complex activity?

Clearly, the focus of this work solely upon seven transmembrane region spanning receptors is a limitation, particularly given changes in receptor tyrosine kinase (RTK) and integrin signalling (unpublished observation) and the possibility of crosstalk between GPCR and RTK/integrin signal transduction mechanisms. The arbitrary picking of Q values, based upon the desire to obtain a “workable” number of hits means that some potentially significant GOIs are excluded from this framework, while others may be inappropriately included. Lastly, the pigmented neurons taken from PD patient samples are the neurons that are resistant to death, potentially making them a survivor subset of the original population rather than a true representation of them. Counter to the survivor bias argument, the broad-based transcriptomic changes in the PD nigral volume do suggest a profound change in nigral microenvironment signalling to pigmented neurons, which seems likely to impact pigmented neuron gene expression.

In conclusion, this work takes existing transcriptomic data and breaks it into nigral volume, pigmented neuron and striatal tissue components to reveal the likely signal transduction process activated by the most abundant signals and G-protein coupled receptors (by transcript). Ultimately, this work shows that while there are many questions relating to signal pathway activation and cross-talk within pigmented neurons, there are two paracrine/autocrine pathways involving DKK3 and dopamine-Gα_o_ whose impacts may, along with paracrine CX3CL1 signalling to microglia, be critical to pigmented neuron survival. At the therapeutic level, the partial restoration or enhancement of a single feedback pathway appears unlikely to significantly promote long-term neuronal survival; for example, MAO-B inhibitors show equivocal effects regarding altering Parkinson’s disease progression [260]. It would, however, be interesting to assess JUND/STAT3 signal modulators or the impact that DKK3- and fractalkine-mimetics have, in combination with dopamine-restoration, as potential therapeutic options for Parkinson’s disease.

## Supporting information

S1 Fig Data set variability as a function of gene abundance. The figure shows the standard deviation (SD) of both pigmented neuron and whole nigral tissue log_2_ differential expression analysis (vertical axis) and gene abundance (expressed as log_2_ transcript per million, TPM, horizontal axis). The nigral volume data shows a linear negative correlation between SD and TPM while the pigmented neuron data shows a one phase decay curve fit (P < 0.0001) in preference to linear regression (F = 17.26, df = 1,2266). The data sets also show a significant difference (P < 0.0001) in variances (F = 4.59, df = 2378, 2709).
(TIF)

S1 Table Summary of data sets, platforms and tissue/cells used. Columns include GSE, whether the study was microarray (platform id) or next generation sequencing (NGS). Type of tissue used and associated reference.
(XLSX)

S2 Table Mean transcript per million (TPM) and differential gene expression analysis (DEA) in pigmented neurons and in the three tissues, substantia nigra, caudate nucleus and putamen. For each cell or tissue data set the first four columns show genes of interest (GOI), average of the control data TPM (log_2_), standard error of the (log_2_) average (SEM) and n values of the control data sets. The next five columns show average of the differential expression analysis (DEA, log_2_), standard error of the (log_2_) average (SEM), n values, P value and an indication of whether that P value is significant with respect to the false discovery rate analysis (Sig?); if that cell is blank the P value is not significant. Within the manuscript the DEA analysis is restricted to genes with n values above 45% of the total number of samples in the data set, although lower “n” value data is shown in the table. Blank cells (or "0") associated with a GOI, for example pigmented neuron Adhesion G Protein-Coupled Receptor D_2_ (*ADGRD2*), indicate that that gene was either not identified in the probe sets or found in a particular set of samples.
(XLSX)

S3 Table Curated list of G-protein coupled receptor genes expressed in pigmented neurons and ranked by transcript per million values. The columns show receptor (GPCR) along with measures of transcript abundance (TPM) and, for the first 34 receptors, primary signal transduction mechanisms (from https://www.guidetopharmacology.org/GRAC).
(XLSX)

S4 Table ChEA3 analysis of changes in significantly elevated transcript expression in pigmented neurons. ChEA3 analysis reveals a list 11 transcription factors that may regulate a majority of the nine significantly elevated transcripts with TPM > 2. ChEA3 Score and Rank indicate the relative strength of association between submitted GOIs and TF.
(XLSX)
