# Supplementary figures and images for: G-protein coupled receptor, signal and signal-transduction related transcript mapping in nigral volume and pigmented neurons reveals a potential deficit of nigral feedback signals associated with Parkinson’s disease
Source: PLoS One. 2026 Jul 24;21(7):e0352503. doi: 10.1371/journal.pone.0352503 (PMC13399538; doi:10.1371/journal.pone.0352503)

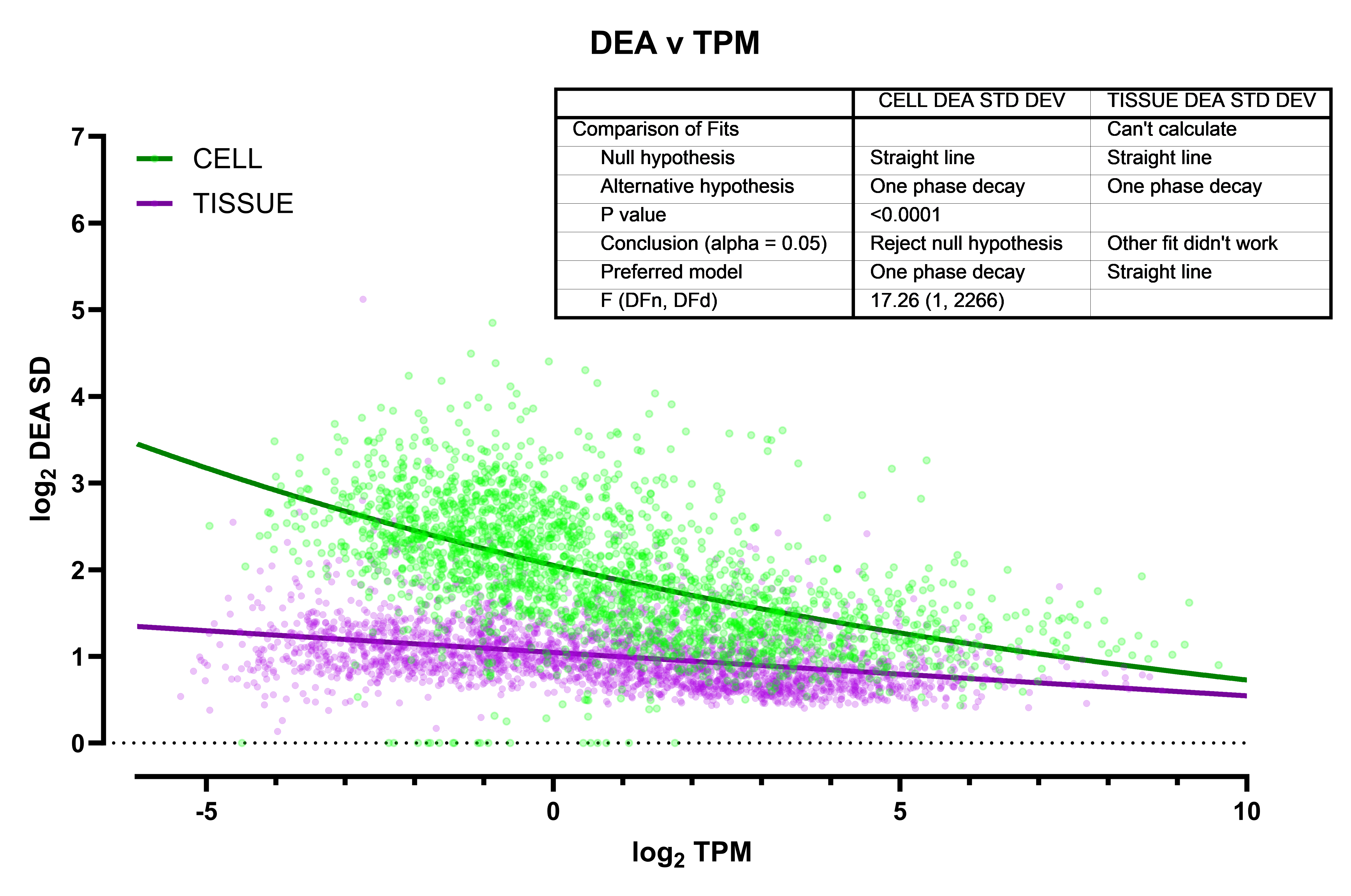

Supplement: S1 Fig — The figure shows the standard deviation (SD) of both pigmented neuron and whole nigral tissue log2 differential expression analysis (vertical axis) and gene abundance (expressed as log2 transcript per million, TPM, horizontal axis). The nigral volume data shows a linear negative correlation between SD and TPM while the pigmented neuron data shows a one phase decay curve fit (P < 0.0001) in preference to linear regression (F = 17.26, df = 1,2266). The data sets also show a significant difference (P < 0.0001) in variances (F = 4.59, df = 2378, 2709). (TIF) [file pone.0352503.s005.tif]
